# Blocking tombusvirus replication through the antiviral functions of DDX17-like RH30 DEAD-box helicase

**DOI:** 10.1371/journal.ppat.1007771

**Published:** 2019-05-28

**Authors:** Cheng-Yu Wu, Peter D. Nagy

**Affiliations:** Department of Plant Pathology, University of Kentucky, Lexington, Kentucky, United States of America; University of California, Davis Genome Center, UNITED STATES

## Abstract

Positive-stranded RNA viruses replicate inside cells and depend on many co-opted cellular factors to complete their infection cycles. To combat viruses, the hosts use conserved restriction factors, such as DEAD-box RNA helicases, which can function as viral RNA sensors or as effectors by blocking RNA virus replication. In this paper, we have established that the plant DDX17-like RH30 DEAD-box helicase conducts strong inhibitory function on tombusvirus replication when expressed in plants and yeast surrogate host. The helicase function of RH30 was required for restriction of tomato bushy stunt virus (TBSV) replication. Knock-down of RH30 levels in *Nicotiana benthamiana* led to increased TBSV accumulation and RH30 knockout lines of *Arabidopsis* supported higher level accumulation of turnip crinkle virus. We show that RH30 DEAD-box helicase interacts with p33 and p92^pol^ replication proteins of TBSV, which facilitates targeting of RH30 from the nucleus to the large TBSV replication compartment consisting of aggregated peroxisomes. Enrichment of RH30 in the nucleus via fusion with a nuclear retention signal at the expense of the cytosolic pool of RH30 prevented the re-localization of RH30 into the replication compartment and canceled out the antiviral effect of RH30. *In vitro* replicase reconstitution assay was used to demonstrate that RH30 helicase blocks the assembly of viral replicase complex, the activation of the RNA-dependent RNA polymerase function of p92^pol^ and binding of p33 replication protein to critical *cis*-acting element in the TBSV RNA. Altogether, these results firmly establish that the plant DDX17-like RH30 DEAD-box helicase is a potent, effector-type, restriction factor of tombusviruses and related viruses. The discovery of the antiviral role of RH30 DEAD-box helicase illustrates the likely ancient roles of RNA helicases in plant innate immunity.

## Author summary

Positive-stranded RNA viruses are important and emerging pathogens that greatly depend on the host during infection. The host uses conserved innate and cell-intrinsic restriction factors as a first line of defense to combat viruses. Among the most intriguing host restriction factors are the family of DEAD-box RNA helicases, which can function as viral RNA sensors or directly as effectors by inhibiting RNA virus replication. RNA helicases are involved in cellular metabolism and perform RNA duplex unwinding and remodeling of RNA-protein complexes in cells. The authors demonstrate that the plant DDX17-like RH30 DEAD-box helicase acts as a strong restriction factor of tombusviruses by blocking multiple steps in the viral replication process. Overall, the findings presented open up a new avenue based on DEAD-box RNA helicases to improve the resistance of plants against viral infections.

## Introduction

Positive-stranded (+)RNA viruses replicate inside cells and depend on many co-opted cellular factors to complete their infection cycle. These viruses build elaborate membranous viral replication compartments, consisting of viral replication proteins, viral RNAs and recruited host factors, in the cytosol of the infected cells. The hijacked host factors participate in all steps of RNA virus replication, including the assembly of membrane-bound viral replicase complexes (VRCs), viral RNA-dependent RNA polymerase (RdRp) activation and viral RNA synthesis. The growing list of co-opted host factors facilitating VRC assembly includes translation initiation and elongation factors, protein chaperones, RNA-modifying enzymes, SNARE and ESCRT proteins, actin network, and lipids [1–9]. Many (+)RNA viruses extensively rewire metabolic pathways, remodel subcellular membranes and take advantage of intracellular trafficking.

The host utilizes cellular proteins to sense viral pathogenicity factors and block virus replication with the help of cell-intrinsic restriction factors (CIRFs) as an early line of defense [2,10–12]. These CIRFs can be part of the innate immune responses and used for antiviral defense as sensors or effectors [13–16]. The identification and characterization of the many CIRFs against different viruses is still in the early stages.

Viral RNA replication is intensively studied with *Tomato bushy stunt virus* (TBSV), a tombusvirus infecting plants, based on yeast (*Saccharomyces cerevisiae*) surrogate host [17–19]. Expression of the two TBSV replication proteins, termed p33 and p92^pol^, and a replicon (rep)RNA leads to efficient viral replication. p92^pol^ is the RdRp [20,21], whereas the more abundant p33 is an RNA chaperone. P33 functions in RNA template selection and recruitment and in the assembly of VRCs within the replication compartment [21–26].

TBSV, which does not code for its own helicase, usurps several yeast and plant ATP-dependent DEAD-box RNA helicases as host factors promoting TBSV RNA replication. The yeast DDX3-like Ded1p and the p68-like Dbp2p, and the plant DDX3-like RH20, DDX5-like RH5 and the eIF4AIII-like RH2 DEAD-box proteins were shown as pro-viral factors, which affect plus- and minus-strand synthesis, maintenance of viral genome integrity and RNA recombination in TBSV [27–29].

DEAD-box helicases are the largest family of RNA helicases and are known to be involved in cellular metabolism [30–32], and affect responses to abiotic stress and pathogen infections [33–35]. They function in unwinding of RNA duplexes, RNA folding, remodeling of RNA-protein complexes, and RNA clamping [36]. They have no unwinding polarity and can open up completely double-stranded RNA regions, however, unlike many other helicases, DEAD-box helicases do not unwind RNA duplexes based on translocation on the RNA strand. Instead, DEAD-box helicases directly load on duplexes and open up a limited number of base pairs. Strand separation within the duplexes is not coordinated with ATP hydrolysis, which is used for enzyme dissociation from the template. This unwinding mode is termed local strand separation [36,37]. DEAD-box helicases also affect RNA virus replication [38–41], and viral translation [42,43]. In case of plant viruses, turnip mosaic virus and brome mosaic virus have been described to co-opt cellular DEAD-box helicases for proviral function in translation or replication [39,42,44]. Altogether, cellular helicases are important co-opted host factors for several viruses, playing critical roles in virus-host interactions.

However, cellular RNA helicases also act as antiviral restriction factors, including functioning as viral RNA sensors (e.g., Dicer or RIG-I) or directly inhibiting RNA virus replication as effectors [45–47]. For example, DDX17 restricts Rift Valley fever virus [48], while DDX21 helicase inhibits influenza A virus and DDX3 blocks Dengue virus infections [49–52]. Thus, the emerging picture is that host helicases are important for the host to restrict RNA virus replication, but the mechanism of their activities or substrates are not well characterized.

In this work, we find that the plant DDX17-like RH30 DEAD-box helicase plays a strong restriction factor function against tombusviruses and related plant viruses. RH30 DEAD-box helicase is expressed in all plant organs, but its cellular function is not known yet [53]. We find that RH30 is re-localized from the nucleus to the sites of tombusvirus replication via interacting with the TBSV p33 and p92^pol^ replication proteins. Several *in vitro* assays provide evidence that RH30 inhibits tombusvirus replication through blocking several steps in the replication process, including VRC assembly, viral RdRp activation and the specific interaction between p33 replication protein and the viral (+)RNA. RH30 knockout lines of *Arabidopsis* supported increased accumulation level for the related turnip crinkle virus, confirming the restriction factor function of RH30 against a group of plant viruses. This is the first identification and characterization of a plant helicase with an effector type restriction factor function against plant viruses. Since plant genomes codes for over 100 RNA helicases, it is likely that additional helicases have CIRF function against plant viruses.

## Results

### The host RH30 RNA helicase is a potent restriction factor of tombusvirus replication in yeast and plants

**Fig 1:**
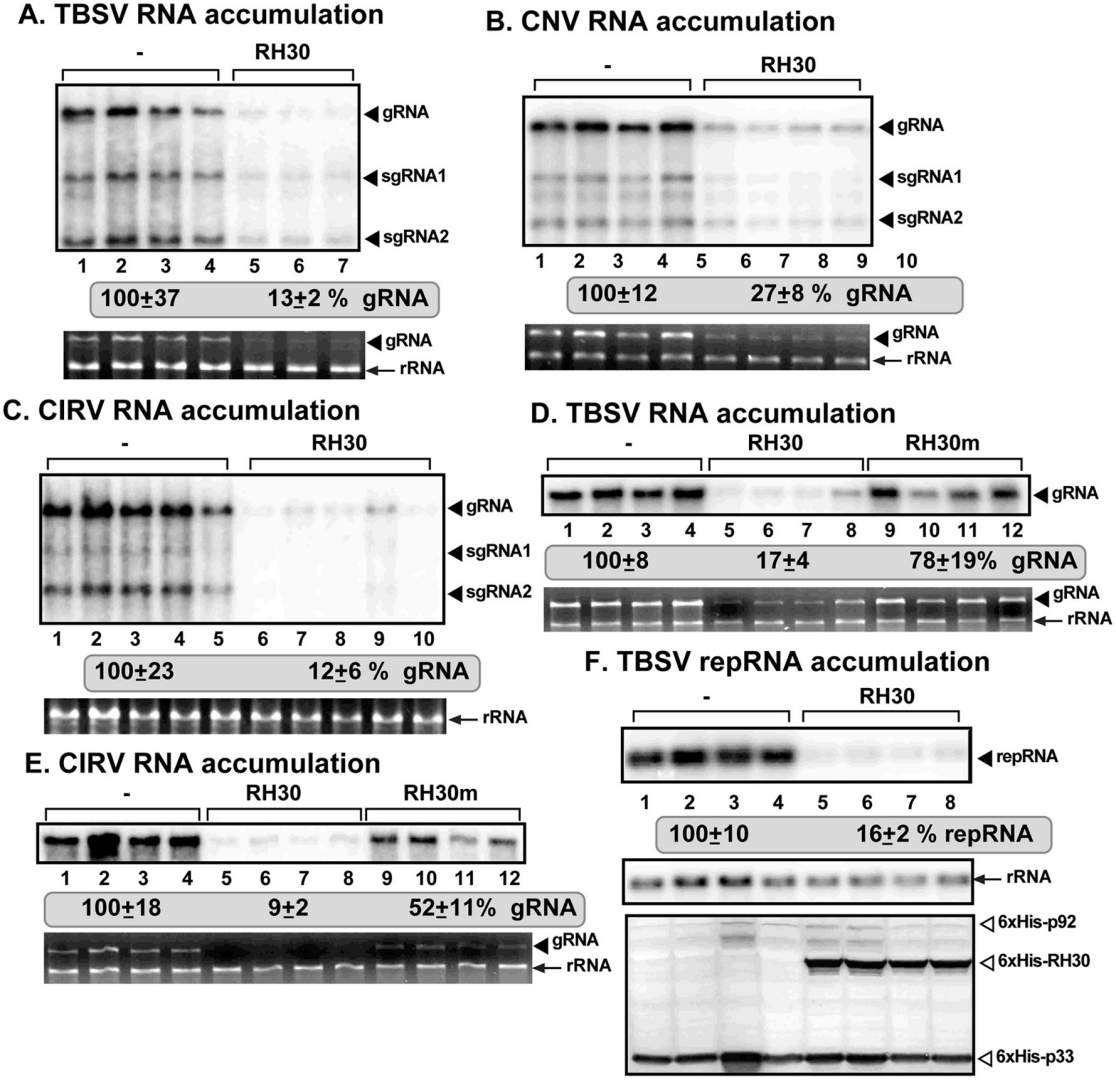
Expression of AtRH30 DEAD-box helicase inhibits tombusvirus genomic (g)RNA replication in *N*. *benthamiana* plant and in yeast surrogate host. *N*. *benthamiana* plants expressing AtRH30 were inoculated with (A) TBSV, (B) CNV, (C) CIRV, respectively. Top panel: Northern blot analyses of tombusvirus gRNA using a 3’ end specific probe shows reduced accumulation of gRNA and subgenomic RNAs in plants expressing RH30 than in control plants. Bottom panel: Ethidium-bromide stained gel shows 18S ribosomal RNA as a loading control. (D-E) Expression of the helicase core mutant of RH30 (RH30m, F_416_L) inhibited TBSV or CIRV replication, respectively, to a lesser extent, demonstrating the requirement of the helicase/ATPase function of RH30 for its full virus restriction function. See further details in panel A. Each experiment was repeated at least three times. (F) Expression of RH30 inhibits TBSV replication in yeast. Top panel: Northern blot analysis of TBSV repRNA using a 3’ end specific probe shows reduced accumulation of repRNA in WT yeast strain expressing RH30. Viral proteins His_6_-p33 and His_6_-p92^pol^ were expressed from plasmids from the *CUP1* promoter, while DI-72(+) repRNA was expressed from the *GAL1* promoter. His_6_-RH30 was expressed from a plasmid. Middle panel: Northern blot with 18S ribosomal RNA specific probe was used as a loading control. Bottom images: Western blot analysis of the level of His_6_-p33, His_6_-p92^pol^ and His_6_-RH30 with anti-His antibody.

To test if the host RH30 RNA helicase could affect tombusvirus replication, we expressed the *Arabidopsis* RH30 using agroinfiltration in *Nicotiana benthamiana* plants. Interestingly, expression of AtRH30 blocked TBSV replication by ~90% in the inoculated leaves (Fig 1A). The closely-related cucumber necrosis virus (CNV), which also targets the peroxisomal membranes for VRC formation, was also inhibited by ~4-fold through the expression of AtRH30 (Fig 1B). Replication of another tombusvirus, carnation Italian ringspot virus (CIRV), which builds the replication compartment using the outer membranes of mitochondria, was inhibited by ~9-fold by the transient expression of AtRH30 in *N*. *benthamiana* (Fig 1C).

To test if RH30 was also effective against TBSV when expressed in yeast cells, we launched the TBSV repRNA replication assay in wt yeast by co-expressing the viral components with RH30. After 24 h of incubation, TBSV repRNA analysis revealed strong inhibition of viral replication by RH30 expression (Fig 1F), suggesting that RH30 is a highly active inhibitor against TBSV replication even in a surrogate host.

To learn if the putative helicase function of RH30 is required for its cell intrinsic restriction factor (CIRF) function, we expressed a motif IV helicase core mutant of RH30(F_416_L) in *N*. *bentamiana* via agroinfiltration. Mutation of the highly conserved F residue within the helicase core domain (see S1 Fig) has been shown to greatly decrease both ATP binding/hydrolysis and strand displacement activities in Ded1 and other DEAD-box helicases [54]. Northern blot analysis revealed the lack of inhibition of TBSV replication, and only partial inhibition of CIRV replication by RH30(F_416_L) (Fig 1D and 1E, lanes 9–12). Thus, we suggest that the full helicase/ATPase function of RH30 is required for its CIRF function against tombusviruses.

**Fig 2:**
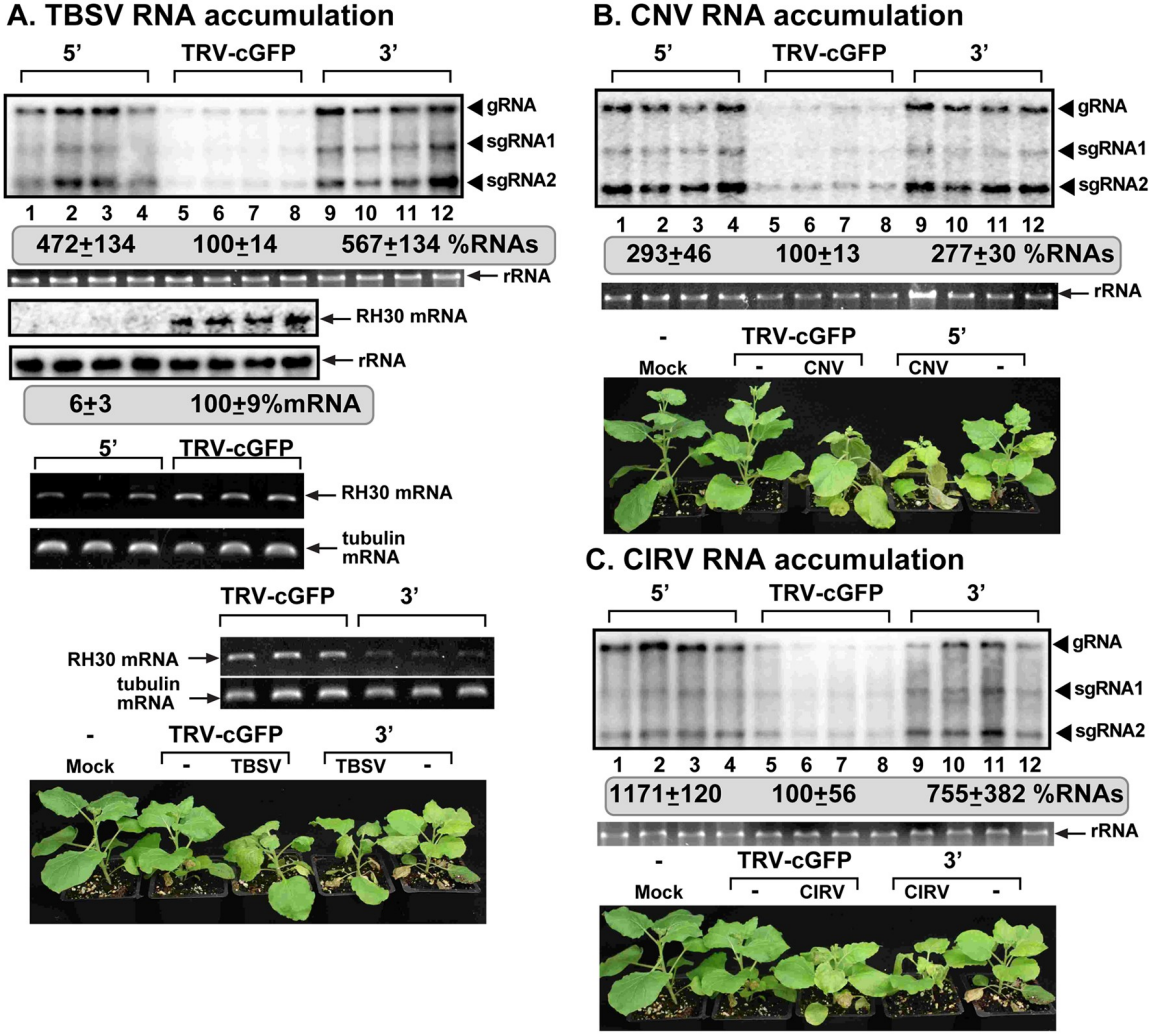
Knockdown of NbRH30 gene expression leads to enhanced tombusvirus replication in *N*. *benthamiana* plants. (A) Top panel: Accumulation of the TBSV genomic (g)RNA and sgRNAs in RH30-silenced *N*. *benthamiana* plants 1.5 days post-inoculation (dpi) was measured by Northern blot analysis. Inoculation of TBSV gRNA was done 12 days after silencing of *RH30* expression. VIGS was performed via agroinfiltration of tobacco rattle virus (TRV) vector carrying 5’ or 3’-terminal NbRH30 sequences, whereas as a control, 3’-terminal GFP sequences. Second panel: Ribosomal RNA is shown as a loading control in an ethidium-bromide stained agarose gel. Third panel: Northern blot analysis shows the knock-down level of NbRH30 mRNA in the silenced and control plants. Fourth panel: Northern blot analysis shows 18S ribosomal RNA as a loading control. Fifth and seventh panels: RT-PCR analysis of NbRH30 mRNA level in the silenced and control plants. Sixth and eighth panels: RT-PCR analysis of *TUBULIN* mRNA level in the silenced and control plants. Each experiment was repeated. Bottom panel: Accelerated and more severe TBSV-induced symptom development is observed in *RH30*-silenced *N*. *benthamiana* plants as compared with the control plants. Note the mild growth defect phenotype in *RH30*-silenced *N*. *benthamiana* plants. The picture was taken 5 dpi. (B-C) Top panel: Accumulation of the CNV or CIRV gRNA in RH30-silenced *N*. *benthamiana* plants 2 days post-inoculation (dpi) was measured by Northern blot analysis. See further details in panel A.

VIGS-based silencing of the endogenous RH30 in *N*. *benthamiana* led to ~5-fold, ~3-fold and ~11-fold increased accumulation of TBSV, CNV and CIRV, respectively, in the inoculated leaves (Fig 2). The leaves of virus-infected and VIGS-treated plants showed severe necrotic symptoms earlier and died earlier than the control plants (i.e., TRV-cGFP treatment) in case of all three tombusvirus infections (Fig 2). On the other hand, the VIGS-treated plants became only slightly smaller than the TRV-cGFP treated control plants (Fig 2). Based on these and the RH30 over-expression data, RH30 DEAD-box helicase seems to act as a major restriction factor against tombusviruses in plants and yeast.

### RH30 DEAD-box helicase is re-localized into the tombusvirus replication compartment in plants

**Fig 3:**
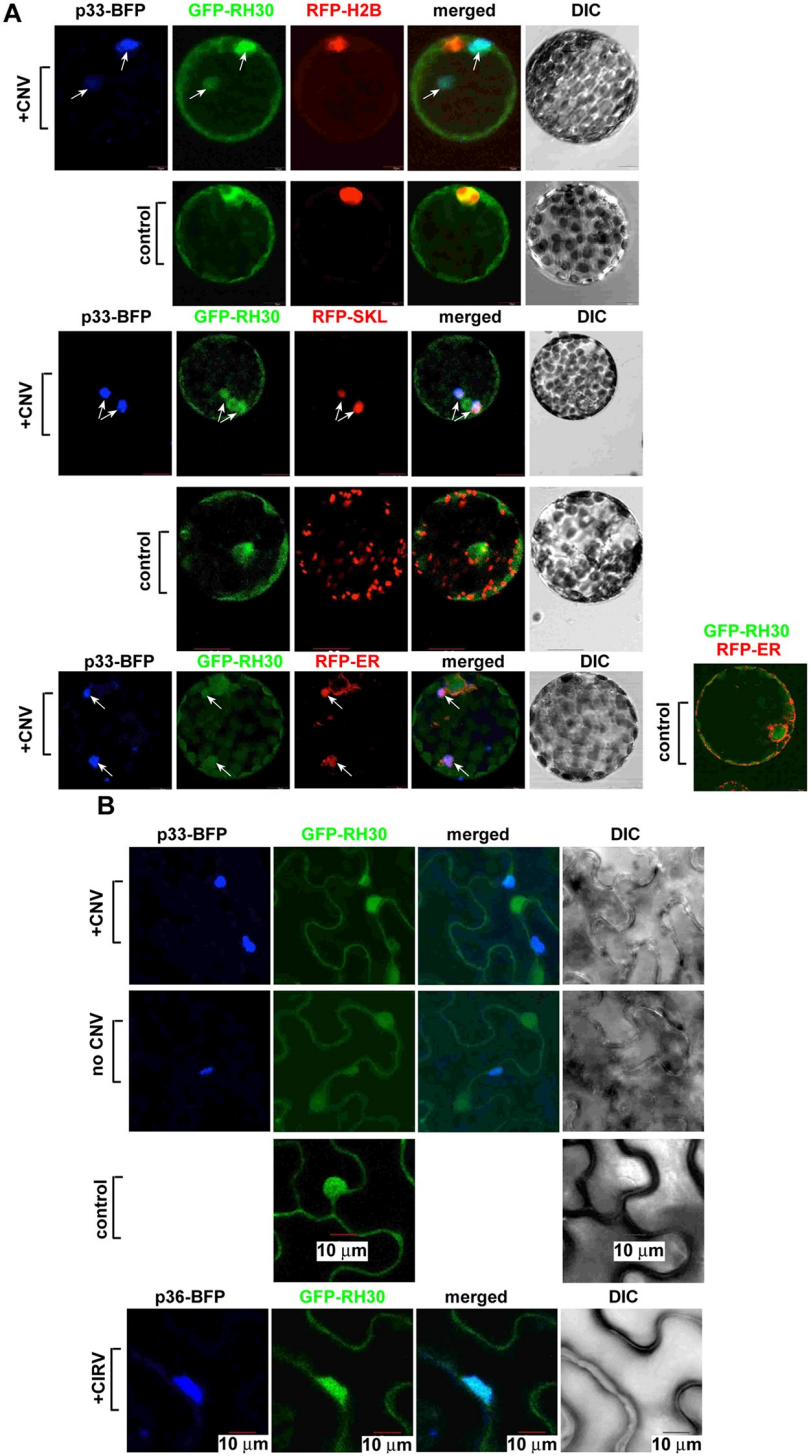
Confocal microscopy shows the retargeting of the mostly nuclear RH30 into the large replication compartment in plant protoplasts and whole plants infected with CNV. (A) Most of RH30 is re-targeted into the replication compartment marked by the BFP-tagged p33 replication protein (pointed by arrows) in *N*. *benthamiana* protoplasts. Second panel: in the absence of viral components, GFP-tagged RH30 is mostly present in the nucleus, as marked by the histone protein (RFP-H2B). Third panel: The re-targeted GFP-RH30 is present in the viral replication compartment, marked by p33-BFP replication protein and RFP-SKL peroxisomal matrix marker. Arrows point at the viral replication compartment. Fourth panel: RH30 is not co-localized to the peroxisomes in the absence of tombusvirus replication. Fifth panel: The re-targeted GFP-RH30 is partially co-localized with the ER marker within the viral replication compartment, marked by p33-BFP replication protein. The leaves of *N*. *benthamiana plants* (transgenic plants expressing nucleus marker RFP-H2B or ER marker RFP-ER) were agro-infiltrated to express p33-BFP, GFP-RH30, and CNV^20KSTOP^ gRNA. Leaves without the expression of p33-BFP and CNV^20KSTOP^ gRNA were used as controls. The agro-infiltrated leaves were collected to isolate protoplasts for confocal imaging 2.5 days post agro-infiltration. Scale bars represent 10 μm. (B) Confocal microscopy images show co-localization of TBSV p33-BFP or CIRV p36-BFP replication proteins and the GFP-RH30 *in planta*. The large replication compartment was visualized via expression of TBSV p33-BFP or CIRV p36-BFP. Expression of the above proteins from the 35S promoter was done after co-agroinfiltration into *N*. *benthamiana* leaves. The leaves of *N*. *benthamiana plants* were agro-infiltrated to express TBSV p33-BFP or the CIRV p36-BFP, GFP-RH30, and CNV^20KSTOP^ or CIRV gRNAs. Leaves without the expression of p33-BFP or p36-BFP and the viral RNAs were used as controls. The agro-infiltrated leaves were collected for confocal imaging 2.5 days post agro-infiltration. Scale bars represent 10 μm. Each experiment was repeated.

To identify the cellular compartment where RH30 DEAD-box helicase performs its CIRF function, first we used co-localization studies in *N benthamiana* protoplasts co-expressing GFP-RH30, p33-BFP (to mark the site of viral replication) and RFP-tagged H2B, which is a nuclear marker protein. We detected the re-localization of GFP-RH30 into the large p33 containing replication compartment from the nucleus during CNV replication (Fig 3A, top panel versus second panel). Both the p33-BFP and RFP-SKL (a peroxisomal matrix marker) showed the re-localization of GFP-RH30 into the large TBSV replication compartment, which consists of aggregated peroxisomes. Part of the ER is also recruited to the p33 and RH30 containing replication compartment (Fig 3A bottom panel), as shown previously [55,56].

Similar re-localization pattern of RH30 was observed in epidermal cells of whole plants infected with CNV (Fig 3B, top panel versus second panel). The expression of only p33-BFP was satisfactory to recruit the RH30 into the replication compartment (Fig 3B). RH30 was also re-targeted in CIRV-infected *N*. *benthamiana* cells into the p36 and p95^pol^ containing replication compartment (Fig 3B, bottom panel), which consists of aggregated mitochondria [57,58]. Based on these experiments, we propose that the mostly nuclear localized RH30 helicase is capable of entering the tombusvirus replication compartment via interaction with the replication proteins. However, the formation of large tombusvirus-induced replication compartments seemed to be normal in the presence of RH30, indicating the lack of interference with the biogenesis of the replication compartment by RH30.

### Nuclear retention of RH30 DEAD-box helicase blocks its antiviral function in plants

**Fig 4:**
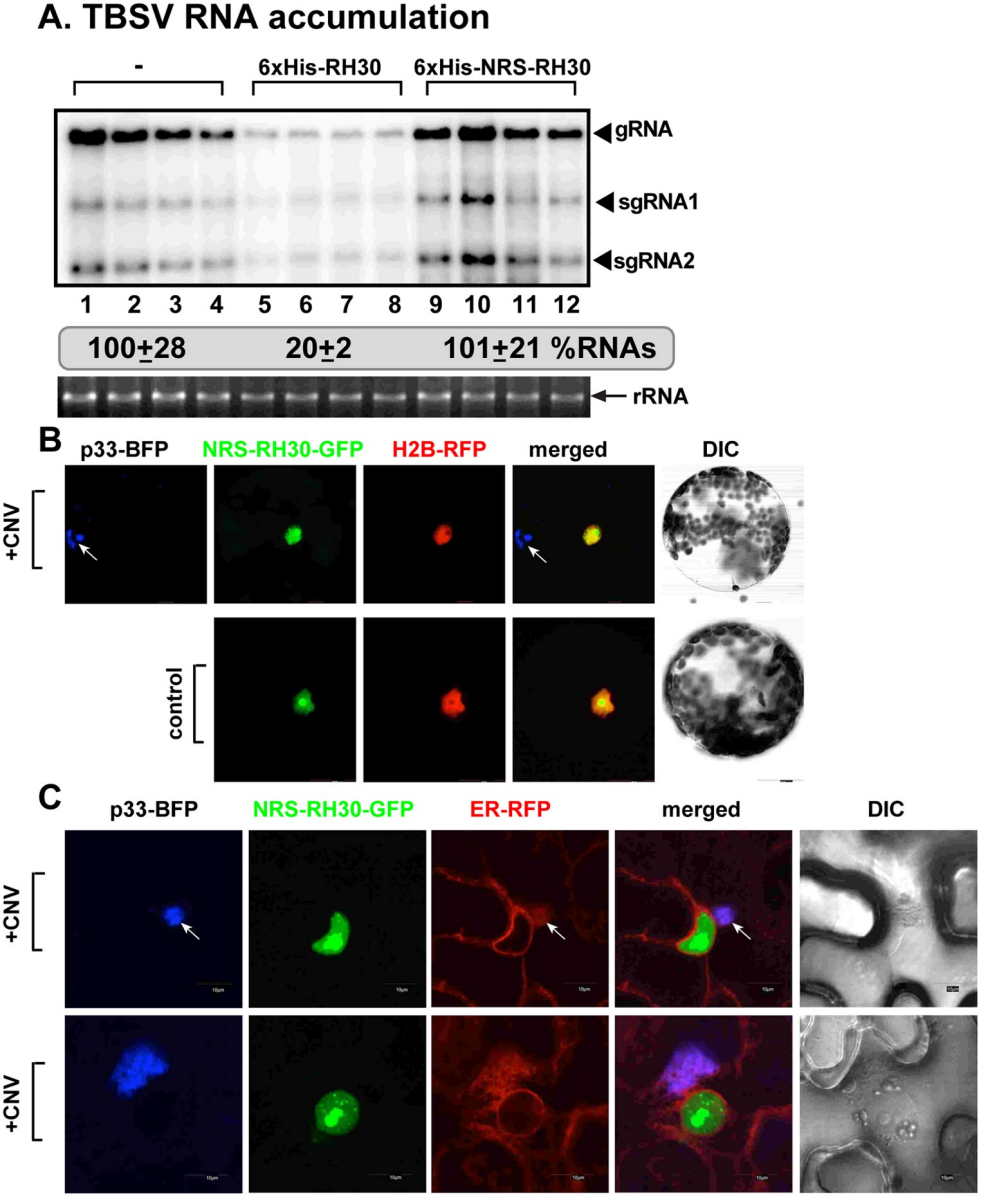
Enrichment of AtRH30 in the nucleus nullifies its antiviral effect against TBSV. (A) Northern blot analysis of TBSV gRNA using a 3’ end specific probe shows lack of inhibition of gRNA accumulation in plants expressing RH30 fused to an NRS. Bottom panel: Ethidium-bromide stained gel to show 18S ribosomal RNA as a loading control. (B) NRS-RH30-GFP is not re-targeted into the replication compartment marked by the TBSV BFP-tagged p33 replication protein (pointed by an arrow) in *N*. *benthamiana* protoplasts. Second panel: in the absence of viral components, NRS-RH30-GFP is present in the nucleus, as marked by the histone protein (H2B-RFP). The leaves of *N*. *benthamiana plants* (transgenic plants expressing nucleus marker RFP-H2B) were agro-infiltrated to express p33-BFP, GFP-RH30, and CNV^20KSTOP^ gRNA. Leaves without the expression of p33-BFP and CNV^20KSTOP^ gRNA were used as controls. The agro-infiltrated leaves were collected to isolate protoplasts for confocal imaging 2.5 days post agro-infiltration. (C) Confocal microscopy images show different localization of TBSV p33-BFP replication protein and NRS-RH30-GFP in *N*. *benthamiana* cells infected with CNV. The large replication compartment was visualized via expression of TBSV p33-BFP. Expression of the above proteins from the 35S promoter was done after co-agroinfiltration into *N*. *benthamiana* leaves. See further details in Fig 3B. Scale bars represent 10 μm. Each experiment was repeated.

To test if the cytosolic localization of RH30 is required for its CIRF function, we fused RH30 with a nuclear retention signal (NRS) [59] to enrich RH30 in the nucleus at the expense of the cytosolic pool of RH30. Interestingly, unlike WT RH30, expression of NRS-RH30 did not result in inhibition of TBSV replication in *N*. *benthamiana* (Fig 4A). Confocal microscopy experiments confirmed that NRS-RH30-GFP is localized exclusively in the nucleus (Fig 4B). Infection of the *N*. *benthamiana* protoplasts with CNV did not result in the re-targeting of NRS-RH30-GFP from the nucleus to the replication compartment visualized via p33-BFP. The nuclear retention of NRS-RH30-GFP was also confirmed in *N*. *benthamiana* epidermal cells infected with CNV or mock inoculated (Fig 4C). Altogether, these experiments demonstrated that re-localization of RH30 helicase from the nucleus to the replication compartment is critical for its CIRF function in plants.

### RH30 helicase interacts with the viral replication proteins in yeast and plants

**Fig 5:**
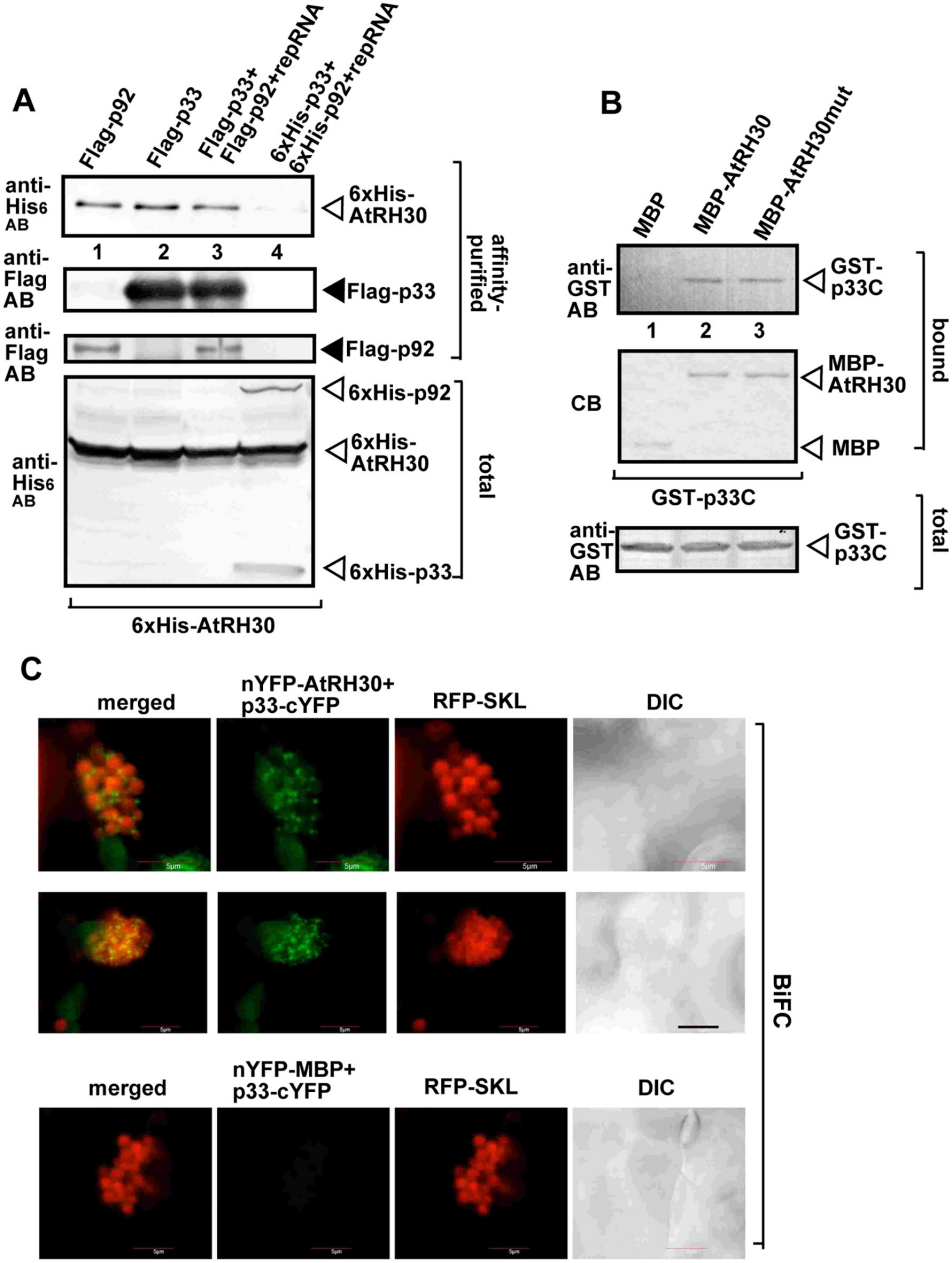
Co-purification of RH30 helicase with the viral replicase from membranous fraction of yeast. (A) Co-purification of His_6_-tagged RH30 with Flag-p33 and Flag-p92^pol^ replication proteins from subcellular membranes. Top panels: Western blot analysis of co-purified His_6_-RH30 (lanes 1, 2, and 3) with Flag-affinity purified replicase, Flag-p33 and Flag-p92^pol^ replication proteins, respectively as shown. His_6_-p33, His_6_-p92^pol^ and His_6_-RH30 were detected with anti-His antibody, while Flag-p33 and Flag-p92^pol^ replication proteins were detected with anti-FLAG antibody. The negative control was from yeast expressing His_6_-RH30, His_6_-p33 and His_6_-p92^pol^ purified in a FLAG-affinity column (lane 4). Bottom panel: blot of total His_6_-p33 and His_6_-p92^pol^ and His_6_-RH30 in the total yeast extracts detected with anti-His antibody. (B) Pull-down assay including TBSV GST-p33 replication protein and the MBP-tagged RH30. Note that we used the soluble C-terminal region of TBSV p33 replication protein, which lacked the N-terminal sequence, including the trans-membrane TM domain. Top panel: Western blot analysis of the captured GST-p33C with the MBP-affinity purified MBP-RH30 or the helicase core mutant of RH30 (RH30mut, F_416_L) was performed with anti-His antibody. The negative control was MBP (lane 1). Middle panel: Coomassie-blue stained SDS-PAGE of the captured MBP-RH30 and MBP. Bottom panel: Western blot analysis of GST-p33C in total E. coli lysates. Each experiment was repeated three times. (C) Interactions between TBSV p33 replication protein and the RH30 helicase was detected by BiFC. The TBSV p33-cYFP replication protein and the nYFP-RH30 and the RFP-SKL peroxisomal marker protein were expressed via agro-infiltration. The merged image shows the efficient co-localization of the peroxisomal RFP-SKL with the BiFC signals, indicating that the interaction between the tombusvirus replication protein and the recruited RH30 helicase occurs in the large viral replication compartments, which consist of aggregated peroxisomes. Scale bars represent 5 μm.

To learn about the tombusviral target of RH30 DEAD-box helicase, we co-expressed the His_6_-tagged RH30 with Flag-tagged p33 and Flag-p92^pol^ replication proteins and the TBSV repRNA in yeast, followed by Flag-affinity purification of p33/p92^pol^ from the detergent-solubilized membrane fraction of yeast, which is known to harbor the tombusvirus replicase [20,60]. Western blot analysis of the affinity-purified replicase revealed the effective co-purification of His_6_-RH30 (Fig 5A, lane 3), suggesting that RH30 targets the VRCs for its CIRF function. Interestingly, His_6_-RH30 was co-purified from yeast co-expressing either Flag-p33 or Flag-p92^pol^ replication proteins (Fig 5A, lanes 1–2), suggesting that RH30 likely directly interacts with the tombusvirus replication proteins in a membranous compartment.

To show direct interaction between RH30 DEAD-box helicase and the TBSV p33 replication protein, we performed pull-down assay with MBP-tagged RH30 and GST-tagged p33 proteins from *E*. *coli*. We found that MBP-RH30 captured GST-p33 protein on the maltose-column (Fig 5B, lane 2), indicating direct interaction between the host RH30 and the viral p33 protein. In the pull-down assay, we used truncated TBSV p33 replication protein missing its N-terminal region including the membrane-binding region to aid its solubility in *E*. *coli* [61]. Interestingly, the helicase core mutant RH30(F_416_L) also bound to p33 replication protein as efficiently as the wt RH30 (Fig 5B, lane 3 versus 2). Altogether, these data suggest that the direct interaction between RH30 host protein and the replication protein of TBSV occurs within the viral protein C-terminal domain facing the cytosolic compartment.

To provide additional evidence that RH30 helicase interacts with the tombusvirus replication protein, we have conducted bimolecular fluorescence complementation (BiFC) experiments in *N*. *benthamiana* leaves. The BiFC experiments revealed interaction between RH30 and the TBSV p33 replication protein within the viral replication compartment, marked by the peroxisomal matrix marker RFP-SKL (Fig 5C). Altogether, these experiments revealed direct interaction between the cellular RH30 DEAD-box helicase and the TBSV p33 replication protein, which results in re-targeting of RH30 into the viral replication compartment.

### RH30 DEAD-box helicase interferes with the assembly of tombusvirus VRCs and activation of p92 RdRp

**Fig 6:**
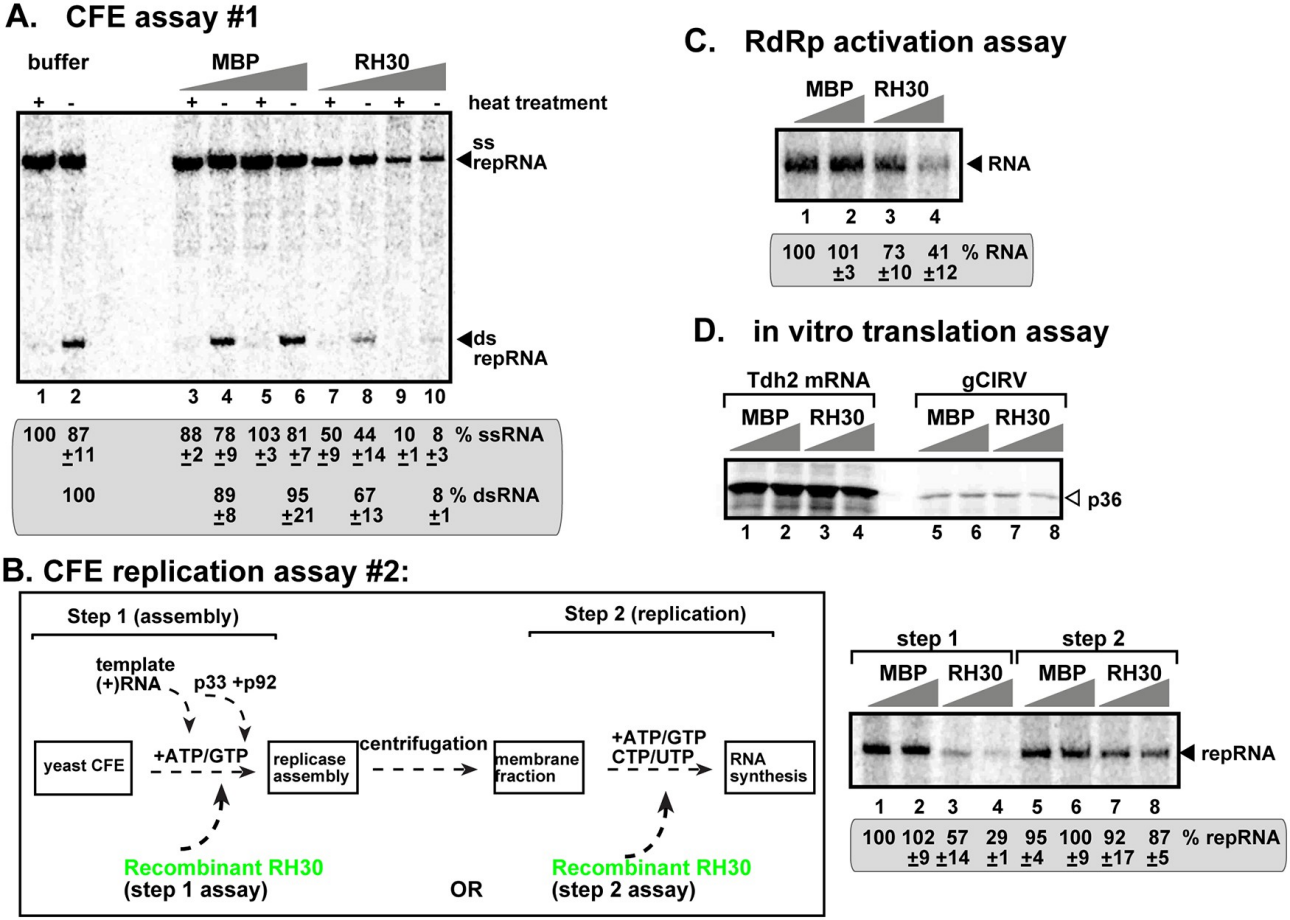
Inhibition of TBSV repRNA accumulation by RH30 in *in vitro* replication assay based on CFE obtained from WT yeast. (A) The purified recombinant tombusvirus p33 and p92 replication proteins from *E*. *coli* were added in combination with the template (+)repRNA to program the *in vitro* tombusvirus replication assay. Increasing amounts (1.9 and 5.7 μM) of purified recombinant MBP-RH30 or MBP, as a control, were added to the reactions. Non-denaturing PAGE shows the accumulation of ^32^P-labeled (+)repRNAs and the dsRNA replication intermediate products made by the reconstituted replicases. Heat treatment, as shown, was applied to demonstrate the dsRNA nature of the replication intermediate. (B) Scheme of the two-step CFE-based *in vitro* replication assay. Step #1 promotes the assembly of the functional tombusvirus replicase, whereas step #2 supports viral RNA synthesis in the presence of all four ribonucleotides. Note that MBP-RH30 or MBP (1.9 and 5.7 μM), as a control, were added to the reactions either at step #1 or step #2, as shown. The ^32^P-labeled TBSV repRNA products of the reconstituted replicases were detected by denaturing PAGE. (C) The *in vitro* RdRp activation assay is based on (+)repRNA and p92-Δ167N RdRp protein in the presence of the soluble fraction of yeast CFE. Purified MBP-RH30 and MBP were added in increasing amounts. Denaturing PAGE analysis of the ^32^P-labeled RNA products obtained in an *in vitro* assay with recombinant p92-Δ167N RdRp. (D) *In vitro* translation assay with wheat germ extract programmed with CIRV gRNA. Purified MBP-RH30 and MBP were added in increasing amounts (1.9 μM and 3.8 μM). The ^35^S-methionine-labeled p36 replication protein translation product is detected by SDS-PAGE. Tdh2 mRNA was used as a control. Each experiment was repeated three times.

To gain insight into the mechanism of CIRF function of RH30 helicase, we affinity-purified the recombinant RH30 and tested its activity *in vitro* in a TBSV replicase reconstitution assay, which is based on yeast cell-free extract [26,62]. Addition of RH30 to the replicase reconstitution assay led to inhibition of TBSV repRNA replication by ~10-fold (Fig 6A, lanes 9–10). The *in vitro* production of double-stranded repRNA replication intermediate was also inhibited by ~10-fold by RH30, indicating that RH30 likely inhibits an early step, such as the VRC assembly during TBSV replication.

We then used a step-wise TBSV replicase reconstitution assay [26,29], in which RH30 was added at different stages of VRC assembly (schematically shown in Fig 6B). RH30 showed significant inhibitory activity when added at the beginning of the TBSV replicase reconstitution assay (Fig 6B, lanes 3–4 versus 1–2). On the contrary, RH30 was ineffective, when added to TBSV replicase reconstitution assay after the VRC assembly step and prior to RNA synthesis (Fig 6B, lanes 7–8). These *in vitro* data support the model that the inhibitory role of RH30 is performed during or prior to the VRC assembly step, but RH30 is ineffective at the latter stages of TBSV replication.

We also utilized an *in vitro* RdRp activation assay based on the purified recombinant TBSV p92 RdRp, which is inactive and requires Hsp70 chaperone and the viral (+)RNA template to become an active polymerase [21]. Addition of the recombinant RH30 helicase strongly inhibited the polymerase activity of the p92 RdRp (Fig 6C), suggesting that RH30 blocks the critical RdRp activation step during tombusvirus replication.

Several RNA helicases are involved in regulation of cellular translation [63]. Therefore, we tested if RH30 affected the translation of tombusvirus genomic RNA, which is uncapped and lacks poly(A) tail [64]. CIRV genomic RNA was used in this *in vitro* assay based on wheat germ extract [65]. Addition of recombinant RH30 to the *in vitro* translation assay inhibited slightly the production of p36 replication protein from the gRNA when RH30 was used in high amount (Fig 6D). The highest amount of RH30 also had minor inhibition on translation of the control Tdh2 mRNA (Fig 6D). Thus, RH30 is unlikely to specifically affect the translation of the tombusvirus RNAs during infection.

### RH30 helicase binds to critical *cis*-acting elements in the viral RNA

**Fig 7:**
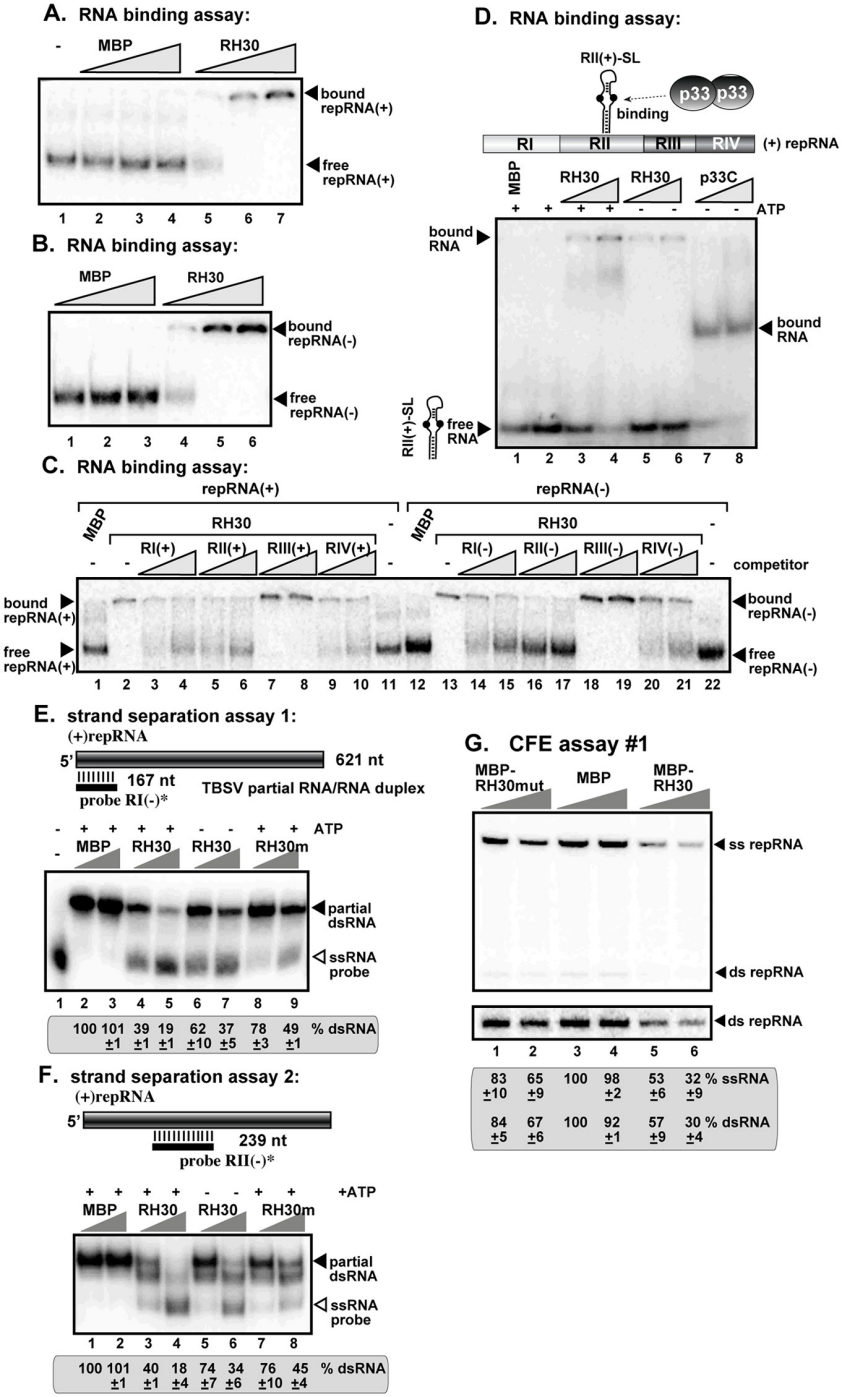
RH30 binds to the RII(+)-SL *cis*-acting element involved in RNA template selection. (A-B) RNA gel mobility shift analysis shows that MBP-RH30 binds to ^32^P-labeled (+)repRNA and (-)repRNA, respectively, *in vitro*. Purified MBP-RH30 or MBP were added in increasing amounts (0.4, 1.9 μM and 5.7 μM) to the assays. The MBP-RH30—^32^P-labeled ssRNA complex was visualized on nondenaturing 5% polyacrylamide gels. Each experiment was repeated at least three times. (C) *In vitro* RNA binding assay with purified RH30. The assay contained 5.7 μM of purified MBP-RH30 or MBP in combination with the ^32^P-labeled (+)repRNA template (~0.1 pmol) or (-)repRNA template (~0.1 pmol) and unlabeled competitor RNAs (2 and 4 pmol) representing one of the four regions of TBSV DI-72 RNA from both RNA strands (see panel D) were used in the competition assay. The MBP-RH30—^32^P-labeled ssRNA complex was visualized on nondenaturing 5% acrylamide gels. Each experiment was repeated at least three times. (D) Schematic representation of the four regions carrying *cis*-acting sequences in the DI-72 (+)repRNA. *In vitro* RNA binding assay with purified MBP-RH30 (1.9 and 5.7 μM) and the ^32^P-labeled RII(+)-SL was performed in the presence or absence of 1 mM ATP. MBP-p33C (1.9 and 5.7 μM) representing the C-terminal soluble portion of TBSV p33 replication protein was used as a positive control, whereas MBP was the negative control. See further details in panel A. (E-F) Top: Schematic representation of the partial RNA/RNA duplexes used in the strand separation assay. The unlabeled template consists of DI-72 (+)repRNA and a short ^32^P-labeled complementary (-)RNA (representing either RI or RII in DI-72), which anneals to the 621 nt DI-72 (+)repRNA. Increasing amounts of purified recombinant MBP-RH30, a helicase core mutant of MBP-RH30m or MBP, as a control, were added to the reactions in the presence or absence of ATP. Bottom: Representative native gel of ^32^P-labeled RNA products after the *in vitro* strand separation assay. Quantification of the partial dsRNA probe was done with a Phosphorimager. This experiment was repeated two times. (G) Increasing amounts (1.9 and 3.8 μM) of purified MBP-fusion protein or MBP (as a control) were added to the *in vitro* CFE assay #1. The ^32^P-labeled RNA products were detected by nondenaturing PAGE. The bottom image shows the contrasted image of the dsRNA bands of the top image.

Since the canonical function of RNA helicases to bind RNA substrates and unwind base-paired structures [36], we tested if RH30 DEAD-box helicase could perform these functions with the TBSV RNA *in vitro*. First, we used gel-mobility shift assay with purified recombinant RH30, which showed that RH30 bound to both the (+) and (-)repRNA (Fig 7A and 7B). Since each of the four regions in the TBSV repRNA contains well-defined *cis*-acting elements, we performed template competition assay with the four regions separately in the presence of recombinant RH30 helicase. This assay defined that the best competitors for binding to RH30 was RII(+) and RII(-), whereas RI(+), RIV(+) and RI(-), RIV(-) also become competitive when added in high amounts (Fig 7C). Because RII(+) contains a critical *cis*-acting stem-loop element, termed RII(+)SL, which is involved in p33-mediated recruitment of the TBSV (+)RNA template [24], and the activation of the p92 RdRp [21], we tested if the purified RH30 could bind to this stem-loop element *in vitro*. Interestingly, RH30 bound to RII(+)SL in the absence of added ATP (Fig 7D). However, the presence of extra ATP enhanced the binding of RH30 to RII(+)SL, suggesting that RH30 binds to RNAs in an ATP-dependent fashion, similar to other DEAD-box helicases [36,54,66]. The control p33 (an N-terminally-truncated, soluble version) bound to RII(+)SL more efficiently and in an ATP-independent manner (Fig 7D), as also shown previously [24]. This highlights the possibility that RH30 and p33 replication protein compete with each other in binding to this critical *cis*-acting element.

To test the RNA helicase function of RH30, we performed strand separation assays, where parts of the TBSV repRNA was double-stranded as shown schematically in Fig 7E and 7F. The RNA helicase activity of RH30 in the presence of ATP was found to efficiently separate the partial dsRNA templates, involving RI and RII sequences (Fig 7E and 7F). RH30 was much less efficient to separate the partial dsRNA templates in the absence of ATP or when we added its helicase core mutant RH30(F_416_L) (Fig 7E, lanes 6–9; 7F, lanes 5–8). It is possible that the residual strand-separation activity of RH30(F_416_L) might come from its RNA binding and RNA chaperone activity with the TBSV RNA substrates. Additional biochemical assays will be needed to test if the partial activity of RH30 in the absence of added ATP is due to the possibly copurified residual ATP bound to RH30.

To test if RH30(F_416_L) helicase core mutant still has antiviral activity, we performed a TBSV replicase reconstitution assay with yeast cell-free extract [26,62]. Addition of RH30(F_416_L) to the replicase reconstitution assay led to minor inhibition of TBSV repRNA replication (Fig 7G, lanes 1–2). Thus, mutation within the helicase core region of RH30 affected its antiviral activity on TBSV replication *in vitro*.

### RH30 helicase inhibits the binding of the viral replication proteins to the template recruitment element in the viral (+)RNA

**Fig 8:**
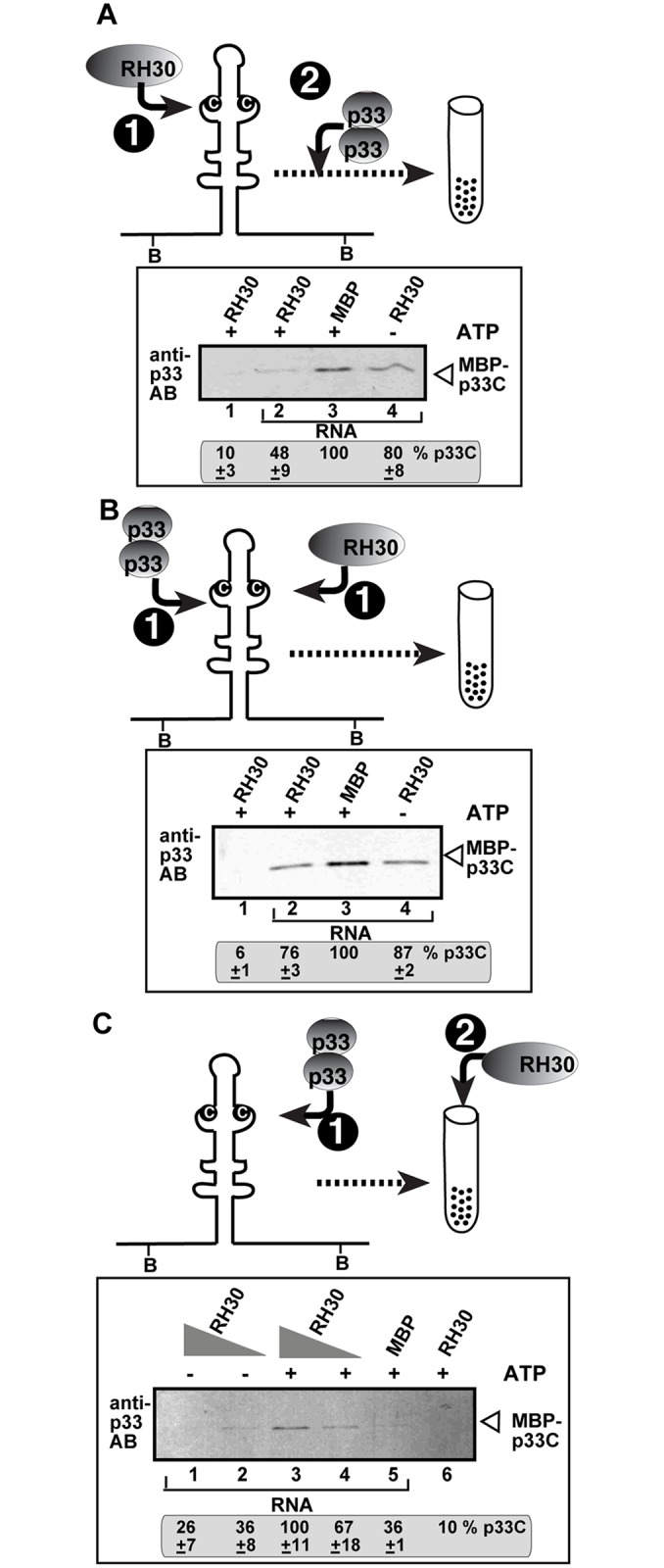
RH30 DEAD-box helicase inhibits the template recruitment by p33 and promotes the release of the viral (+)RNA from p33 replication protein *in vitro*. (A) Top: Scheme of the *in vitro* assay with biotinylated RII(+) RNA from TBSV bound to streptavidin-coated magnetic beads. The scheme shows the order of addition of biotin-labeled RII(+) RNA, MBP-RH30 and MBP-p33C to the *in vitro* assay. The RNA probe and MBP-RH30 was allowed to form an RNP complex for 15 min, followed by addition of MBP-p33C protein, and incubation for 15 min. Then, the biotin-labeled RII(+) RNA—protein complex was captured on streptavidin-coated magnetic beads and washed the beads with a buffer. We eluted the proteins from the beads and measured the amounts of MBP-p33C in the eluates by Western blotting using anti-p33 antibody. Reduced amounts of MBP-p33C in the eluates mean that RH30 prevented the binding of p33C to the viral RNA, likely due to remodelling the RNA structure that could not be recognized by p33 any longer. Nonbiotinylated RNA (lane 1) was used as a control. (B) The scheme shows that the biotin-labeled RII(+) RNA, MBP-RH30 and MBP-p33C were added simultaneously to the *in vitro* assay. See additional details in panel A. (C) Top: The scheme shows that the biotin-labeled RII(+) RNA probe and MBP-p33C was allowed to form an RNP complex for 30 min, followed by capturing the biotin-labeled RII(+) RNA—protein complex on streptavidin-coated agarose beads. Then, we added MBP-RH30 protein with or without ATP, followed by incubation for 15 min and washing the beads with a small amount of buffer. Then, we measured the amount of MBP-p33C in the eluates by Western blotting using anti-p33 antibody. Increased amounts of MBP-p33C in the eluates mean that RH30 displaced p33C from the viral RNA, likely due to remodelling the RNA structure that could not be recognized by p33 any longer. Nonbiotinylated RNA (lane 6) was used as a control. Each experiment was repeated four times.

To further characterize the restriction function of RH30 during tombusvirus replication, we tested if RH30 helicase could inhibit the selective binding of p33 replication protein to the viral RNA template *in vitro*. To this end, we biotin-labeled RII(+) sequence of the TBSV RNA, which represents RII(+)-SL RNA recognition element required for template recruitment into replication by p33 replication protein [24]. Moreover, RII(+)-SL RNA is also essential part of an assembly platform for the replicase complex [67]. The biotin-labeled RII(+) RNA was then pre-incubated with purified RH30 (Fig 8A). Then, purified p33C (the soluble C-terminal region, including the RNA-binding and p33:p33/p92 interaction region of p33 replication protein) was added, which can bind specifically to RII(+)-SL if the hairpin structure with the C•C mismatch in the internal loop was formed [24]. After a short incubation, the biotin-labeled RII(+) RNA was captured on streptavidin-coated magnetic beads. After thorough washing of the streptavidin beads, the proteins bound to the RNA were eluted. Western blot analysis with anti-p33 antibody revealed that RH30 in the presence of ATP inhibited the binding of p33C to RII(+)-SL by 50% (Fig 8A, lane 2 versus lane 3) when compared with the control containing the MBP protein that does not bind to RII(+)-SL [24]. RH30 was less inhibitory of the p33C—RII(+)-SL interaction in the absence of ATP (Fig 8A, lane 4). We also performed the experiments when RH30 and p33C were incubated with biotin-labeled RII(+) RNA simultaneously. Western-blot analysis showed that RH30 was still inhibitory of p33C binding to RII(+)-SL (Fig 8B), but less effectively than above when RH30 was pre-incubated with the RII(+) RNA. These *in vitro* results suggest that one of the mechanisms by which RH30 helicase inhibits tombusvirus replication is to inhibit the binding of p33 to the critical RII(+)-SL RNA recognition element required for template recruitment into replication. This inhibition is likely due to local unwinding RII(+)-SL, because the presence of ATP enhanced the inhibitory effect of RH30.

In another set of experiments, we first incubated biotin-labeled RII(+) RNA with p33C, followed by capturing the RNA-p33 complex with streptavidin-coated magnetic beads and then, the addition of RH30 helicase to the beads (Fig 8C). Here we tested the released p33C from the beads in the eluted fraction by Western blotting. Interestingly, increasing the amounts of RH30 added in the presence of ATP led to the release of p33C from the RII(+) RNA (Fig 8C, lane 3–4), whereas RH30 was less efficient in replacing p33C in the absence of ATP (lanes 1–2). Based on these *in vitro* data, we suggest that RH30 helicase could replace the RNA-bound p33C by likely remodeling the RNA-p33 complex in an ATP-dependent manner.

### RH30 helicase is co-localized with the viral dsRNA replication intermediate within the tombusvirus replication compartment in plants

**Fig 9:**
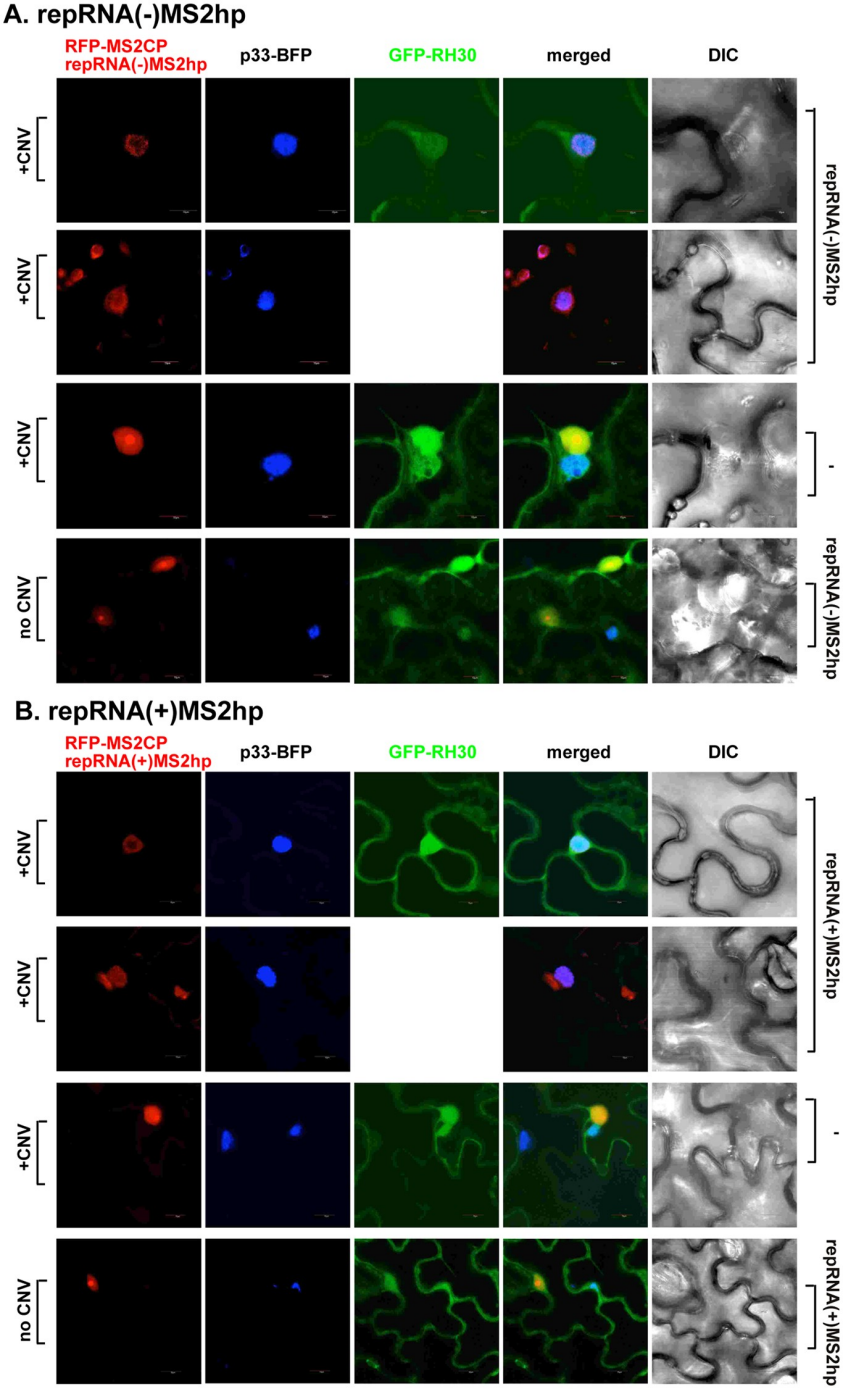
Confocal microscopy shows co-localization of RH30 with the viral repRNAs in whole plants infected with CNV. (A-B) Most of RH30 is re-targeted into the replication compartment where RNA synthesis takes place. The viral (-)repRNA and (+)repRNA carried six copies of the MS2 phage RNA hairpin (MS2hp) recognized by MS2 CP fused with RFP. The replication compartment was also marked by the BFP-tagged p33 replication protein in *N*. *benthamiana*. Note that RFP-MS2CP contains a weak nuclear localization, therefore this protein ends up in the nucleus in the absence of target RNAs in the cytosol. Expression of the above proteins from the 35S promoter was done after co-agroinfiltration into *N*. *benthamiana* leaves. The leaves of *N*. *benthamiana* plants were agro-infiltrated to express TBSV p33-BFP, GFP-RH30, RFP-MS2CP, repRNA(-)MS2hp or repRNA(+)MS2hp and the helper virus CNV^20KSTOP^ gRNA. The repRNA(+)MS2hp consists of the repRNA(+) carrying six copies of *cis*-MS2 hairpin, which can be bound by RFP-MS2CP to show the subcellular localization of repRNA(+). The repRNA(-)MS2hp consists of repRNA(+) carrying six copies of *trans*-MS2 hairpin, which can only be recognized by RFP-MS2CP when viral RNA replication produces the complimentary strand repRNA(-) by the helper virus CNV^20KSTOP^. The absence of transient expression of GFP-RH30, repRNA(+)/(-)MS2hp or CNV^20KSTOP^ were used as controls. The agro-infiltrated leaves were collected for confocal microscopy imaging 3.5 days post infiltration. Scale bars represent 10 μm. Each experiment was repeated.

**Fig 10:**
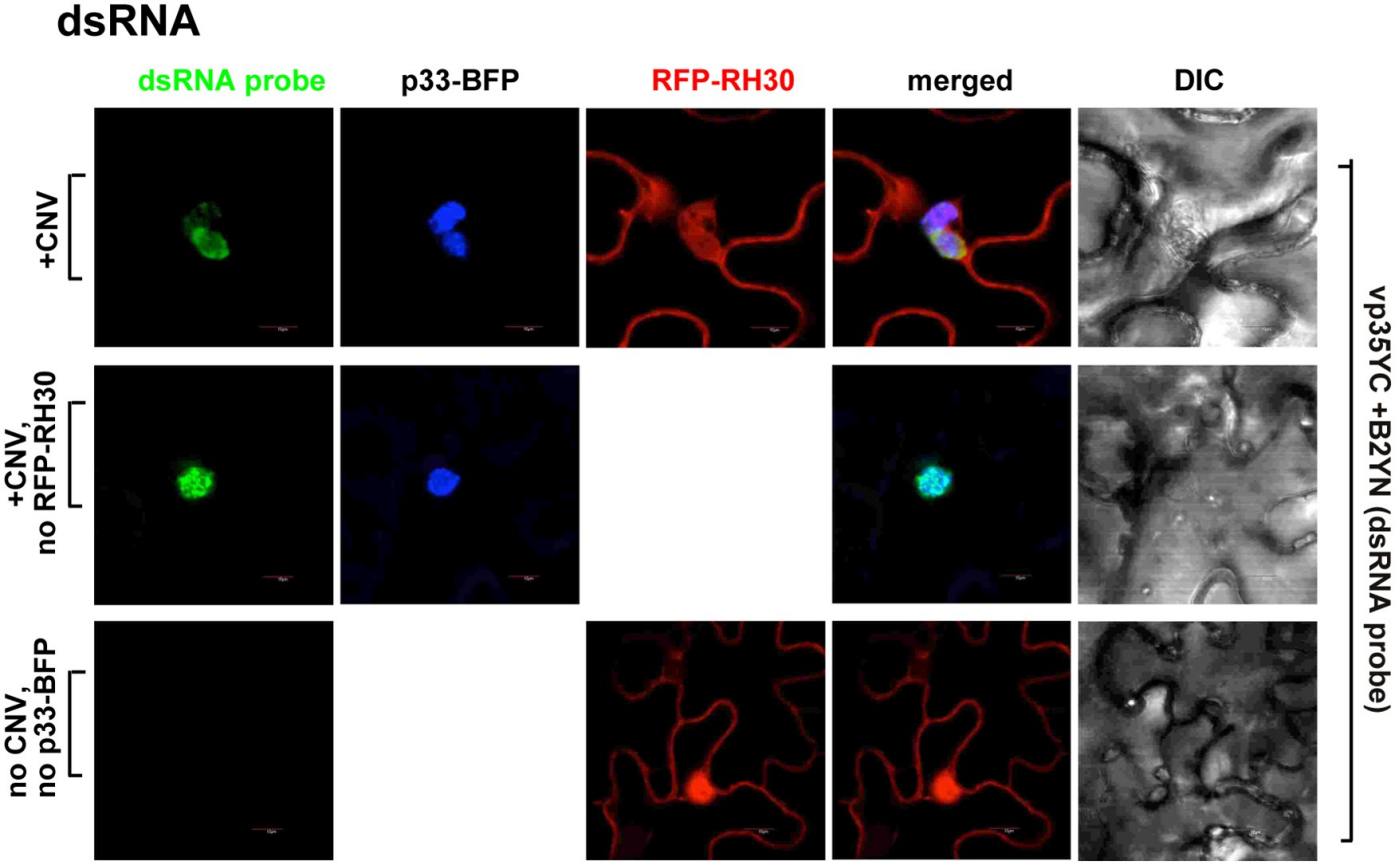
Co-localization of the viral double-stranded gRNA with RH30 in whole plants infected with CNV. The CNV genomic dsRNA replication intermediate was detected via a dsRNA detector assay based on dsRNA binding-dependent fluorescence complementation assay [69]. The assay was performed with two dsRNA binding proteins (i.e., vp35 and B2), which are fused to N- and C-terminal halves of the yellow fluorescence protein (YFP), respectively. Simultaneous binding of the two fusion proteins to the same CNV dsRNA replication intermediate leads to the restoration of YFP fluorescence, allowing the visualization of the viral dsRNA replication intermediate location via confocal microscopy. The dsRNA sensor B2YN and VP35YC plasmids were agro-infiltrated into *N*. *benthamiana* leaves at OD_600_ of 0.15, respectively, together with RFP-RH30 and p33-BFP at OD_600_ of 0.5. CNV infection was initiated via agro-infiltration (OD_600_ of 0.15). Leaves were harvested and then immediately subjected to confocal microscopic analysis 2 days after agro-infiltration. The fluorescence complementation was detected via the GFP channel (excitation/emission: 488nm/500-530nm). Top panel: viral dsRNA replication intermediate is co-localized with RFP-RH30 within the replication compartment, which is marked by TBSV p33-BFP. Middle panel: no expression of RFP-RH30 was used as control. Bottom panel: *N*. *benthamiana* leaves with no viral components expressed were used as control. Expression of the above proteins from 35S promoter was done after co-agroinfiltration into *N*. *benthamiana* leaves. Scale bars represent 10 μm. Each experiment was repeated.

We also tested the localization of RH30 helicase in comparison with the viral repRNA in *N*. *benthamiana*. The TBSV repRNA carried six copies of the RFP-tagged coat protein recognition sequence from bacteriophage MS2 in either plus or minus polarity [68]. CNV served as a helper virus in these experiments. Interestingly, RH30 was co-localized with both (-)repRNA and (+)repRNA, which were present in the replication compartment decorated by the TBSV p33-BFP (Fig 9A and 9B). The RFP signal within the replication compartment was usually weaker when RH30 helicase was expressed, likely due to the inhibitory effect of RH30 on tombusvirus replication. Similar outcome was observed when the viral dsRNA replication intermediate, detected via dsRNA probes [69], was co-localized with RH30 helicase within the replication compartment (Fig 10). These data demonstrate that RH30 helicase relocates to the replication sites where tombusvirus RNA synthesis takes place.

### RH30 DEAD-box helicase inhibits the accumulation of related and unrelated plant and insect viruses in yeast or plants

**Fig 11:**
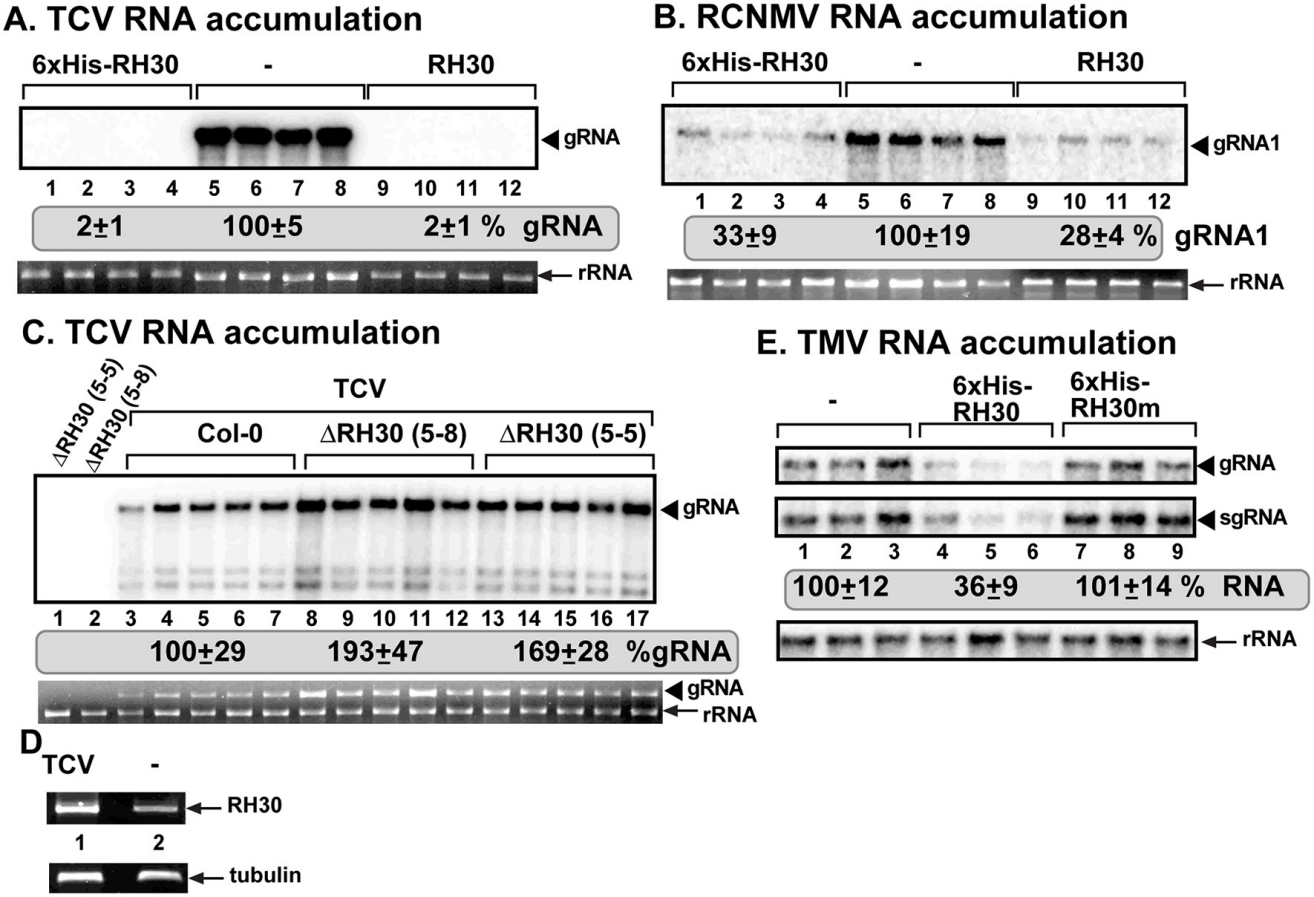
Expression of AtRH30 DEAD-box helicase inhibits TCV and RCNMV genomic (g)RNA replication in *N*. *benthamiana* plants. *N*. *benthamiana* plants expressing AtRH30 were inoculated with (A) TCV, and (B) RCNMV, respectively. Expression of the above proteins from the 35S promoter was done via co-agroinfiltration into *N*. *benthamiana* leaves. Top panel: Northern blot analyses of TCV gRNA and RCNMV RNA1 using 3’ end specific probes show reduced accumulation of TCV gRNA and RCNMV RNA1, respectively, in plants expressing RH30 than in control plants. Bottom panel: Ethidium-bromide stained gel to show 18S ribosomal RNA as a loading control. (C) Increased accumulation level of TCV in *Arabidopsis* RH30 knockout mutants based on Northern blot analysis. Samples in lanes 1 and 2 are from mock- inoculated *Arabidopsis* RH30 knockout mutants. See further details in panel A. (D) Semi-quantitative RT-PCR shows the induction of RH30 mRNA expression in *Arabidopsis* plants infected with TCV when compared the mock-inoculated plants. Each experiment was repeated. (E) Expression of RH30 and its mutant protein together with the cDNA of full-length TMV from the 35S promoter was done via co-agroinfiltration into *N*. *benthamiana* leaves. Top panel: Northern blot analysis of TMV gRNA and subgenomic RNA using a 3’ end specific probe shows reduced accumulation of TMV RNAs in leaves expressing RH30, but not RH30m in comparison with the control plants. Bottom panel: Northern blot analysis shows the 18S ribosomal RNA as a loading control. Each experiment was repeated.

To learn if RH30 has restriction function against additional plant viruses, we tested the effect of RH30 expression on TCV carmovirus and red clover necrosis mosaic virus (RCNMV) dianthovirus, both of which belong to the Tombusviridae family. Expression of AtRH30 in *N*. *benthamiana* plants led to complete block of TCV gRNA accumulation and ~4-fold reduction in RCNMV RNA1 accumulation (Fig 11A and 11B). On the contrary, two separate transgenic RH30 knock-out lines of *Arabidopsis thaliana* supported increased levels of TCV gRNA accumulation by up to 2-fold (Fig 11C).

The Arabidopsis-TCV system was also used to estimate if TCV infection could induce RH30 gene transcription. RT-PCR analysis revealed induction of RH30 mRNA transcription in TCV-infected versus mock-inoculated plants (Fig 11D). All these data are in agreement that RH30 is a strong restriction factor against tombusviruses and related viruses in plants.

To learn if RH30 also has restriction function against an unrelated plant virus, we over-expressed AtRH30 in *N*. *benthamiana* and measured the accumulation of the unrelated tobacco mosaic tobamovirus (TMV). We observed a ~3-fold reduction in TMV RNA accumulation in *N*. *benthamiana* leaves expressing the WT RH30, but not in those leaves expressing the helicase core mutant of RH30(F_416_L) (Fig 11E). Expression of WT RH30, but not that of the RH30(F_416_L) helicase core mutant, also inhibited the accumulation of the insect-infecting Nodamura virus (NoV) by ~3-fold in yeast (S2A Fig). Interestingly, the accumulation of Flock House virus (FHV), an alphanodavirus, which is related to NoV, was only slightly inhibited by the expression of WT RH30 in yeast (S2B Fig). Based on these observations, we suggest that the plant RH30 DEAD-box helicase has a broad-range CIRF activity against several RNA viruses.

## Discussion

DEAD-box RNA helicases are the most numerous among RNA helicases [33,37]. They are involved in all facets of RNA processes in cells. RNA viruses and retroviruses also usurp several DEAD-box helicases to facilitate their replication and other viral processes during infection [70,71]. However, the host also deploys DEAD-box helicases to inhibit RNA virus replication [70,72]. Accordingly, in this work we present several pieces of evidence that the DDX17-like RH30 DEAD-box helicase restricts tombusvirus replication, including the peroxisomal replicating TBSV and CNV and the mitochondrial-replicating CIRV in yeast and plants, and the more distantly related TCV and RCNMV and the unrelated TMV in plants. On the contrary, knock-down of RH30 enhances the replication of these three tombusviruses in *N*. *benthamiana* or the related TCV in RH30 knock-out lines of *Arabidopsis*. On the other hand, the helicase core mutant RH30 can only partially inhibit tombusvirus replication in plants or *in vitro*, suggesting that the helicase function of RH30 is needed for its full antiviral activity.

**Fig 12:**
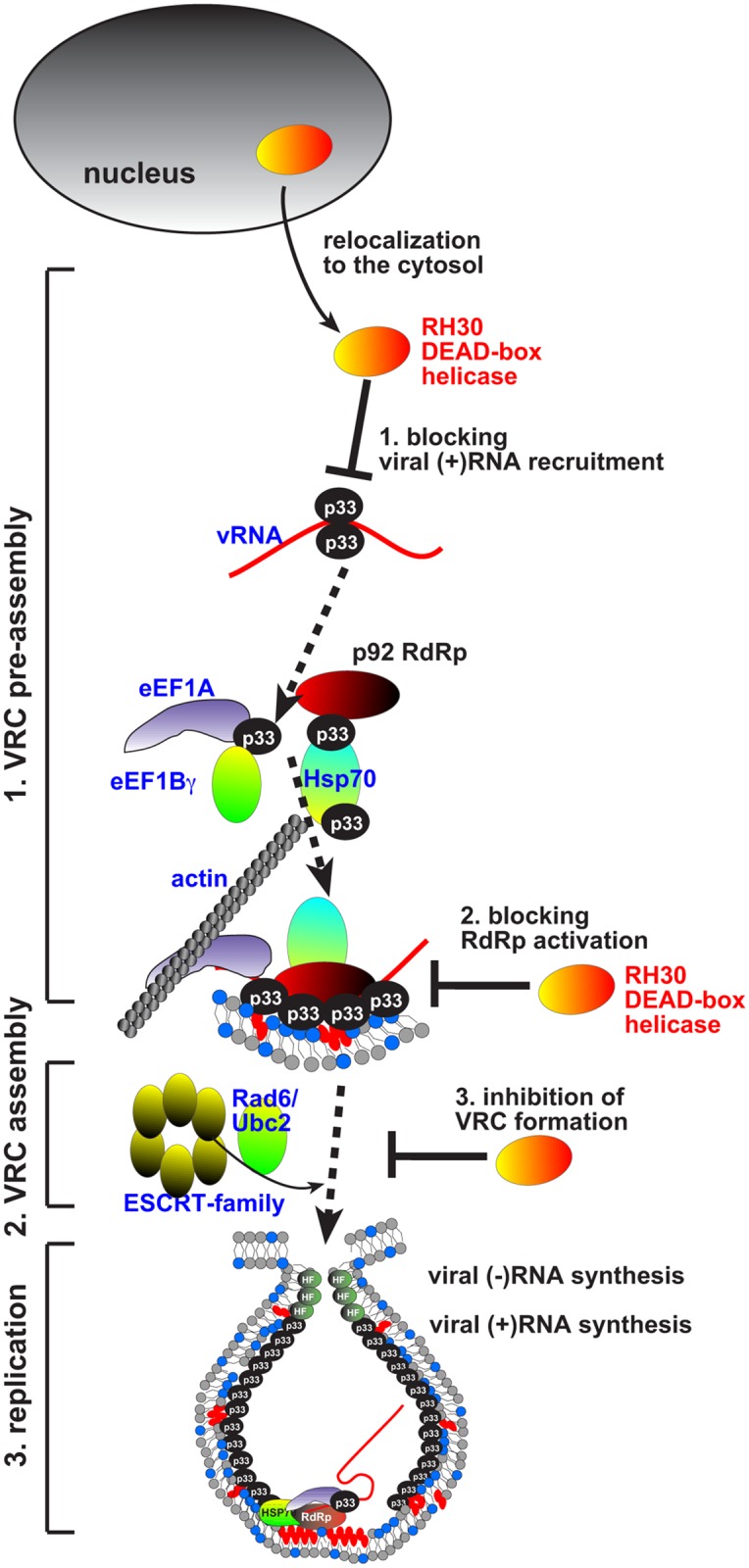
Models showing the antiviral functions of the plant RH30 DEAD-box helicase during TBSV replication. Based on our current and previous data, we propose that the DDX17-like RH30 helicase interferes with several major steps during TBSV replication. First, RH30 interferes with the recruitment of the viral (+)RNA through unwinding RII(+)-SL *cis*-acting RNA element, which specifically binds to p33 replication protein only when the stem-loop structure is formed. Also, RH30 can potentially remodel the p33-(+)RNA complex, thus displacing p33 from the complex. Second: Inhibition of p33-(+)RNA complex formation by RH30 also leads to blocking the activation of the p92 RdRp, which requires the (+)RNA with the stem-loop structure in RII(+)-SL formed. Third, displacing p33 from the p33-(+)RNA complex by RH30 inhibits VRC assembly as well. This is because the stem-loop structure in RII(+)-SL is essential part of the VRC assembly platform. The cytosolic pool of RH30 is essential for the antiviral activity.

How can RH30 restrict TBSV replication? We show that the antiviral RH30 helicase binds to p33 and p92^pol^ replication proteins based on co-purification experiments of the viral replicase complex, a pull down assay, and BiFC in *N*. *benthamiana*. We propose that the interaction of RH30 helicase with the viral replication proteins might be important for the targeting of RH30 into the viral replication compartment (Fig 12). Accordingly, RH30 is recruited into the viral replication compartment from the cytosol and the nucleus based on live imaging in plant cells (Fig 3). The targeting of RH30 into the replication compartment is critical for its antiviral function, because fusion of a nuclear retention signal with RH30, which leads to its enrichment in the nucleus at the expense of the cytosolic pool of RH30, in turn, cancelled out the antiviral effect of RH30. Yeast CFE-based replicase reconstitution assays showed that RH30 acts in the early steps of replication, since both (-) and (+)RNA synthesis was inhibited by RH30 (Fig 6). Moreover, the *in vitro* RdRp activation assay demonstrated that RH30 inhibited the TBSV RdRp activation step during the replication process as well (Fig 6C). In contrast, the CFE-based TBSV replication was not inhibited by RH30 after replicase assembly was completed (see step 2, Fig 6B). These data suggest that RH30 DEAD-box helicase must act at the earliest steps in the replication process to inhibit TBSV replication.

RH30 also binds to the viral RNA, including the 5’ UTR (i.e., RI) and RII internal sequence present within the p92^pol^ coding region (Fig 7). Using *in vitro* interaction and replication assays between RNA-p33 replication protein, we show that RH30 inhibits several steps in tombusvirus replication. These include the RH30-based inhibition of (i) the specific recognition of the critical RII(+)-SL *cis*-acting element in the viral (+)RNA by p33 replication protein, which is absolutely required for template recruitment into VRCs, (ii) the activation of the viral p92 RdRp, and (iii) the assembly of the VRCs [21,26,73]. Moreover, RH30 helicase could disassemble viral RNA-p33 complexes by likely remodeling the RNA structure in an ATP-dependent manner (Fig 8). However, RH30-mediated disassembly of viral RNA-p33 complexes is unlikely to occur after VRC assembly is completed, because RH30 helicase was not an effective restriction factor when added at a late step of TBSV replication (step 2, Fig 6B). We propose that the membrane-bound TBSV VRCs are protecting the viral RNA-p33 complexes by restricting accessibility of the VRC complex to RH30 DEAD-box helicase. Accordingly, we have shown before that the fully-assembled TBSV VRCs are resistant to cellular ribonucleases [74]. Therefore, RH30 helicase might only be able to disassemble viral RNA-p33 complexes before the vesicle-like spherule formation, which is the characteristic structure of the TBSV VRCs in yeast and plants [75]. Altogether, the *in vitro* assays provide plentiful data on the direct inhibitory effect of RH30 helicase on TBSV replication, indicating that RH30 functions as an effector-type, not signaling-type, DEAD-box helicase, which detect viral RNA and send signals to downstream components of the innate immunity network [72]. Future experiments will address if RH30 might have additional mechanisms to restrict tombusvirus replication.

A recently emerging concept in innate immunity is the significant roles of DEAD-box helicases expressed by host cells that greatly reduce virus replication and facilitate combating viruses and making the induced and passive innate immune responses more potent. Many of the identified yeast DEAD-box helicases with restriction functions against TBSV are conserved in plants and mammals. Altogether, the genome-wide screens performed with animal viruses have shown that helicases are the largest group of host proteins affecting RNA virus replication. For example, in case of HIV, the involvement of several cellular helicases has been demonstrated, including DDX17 and DDX3 [71,76,77]. Yet, the functions of the cellular helicases during virus replication are currently understudied.

The emerging pricture in plant-virus interactions, similar to animal-virus interactions, is the diverse roles of various host RNA helicases. Different plant viruses have been shown to co-opt plant RNA helicases for pro-viral functions. These include RH8 and RH9 for potyvirus replication and RH20, RH2 and RH5 for TBSV replication [27–29,39,44,78]. However, this paper shows evidence that a plant DEAD-box helicase, RH30, can also be utilized by host plants for antiviral functions. Thus, in addition to the previously identified Dicer-like RNA helicases [16,79–81], additional plant RNA helicases might function as CIRFs by recognizing plant virus RNAs. The DDX17-like RH30 DEAD-box helicase characterized here opens up the possibility that among the more than 100 helicases of plants, there are additional ones with antiviral functions, serving as effector-type or sensor-like RNA helicases. The discovery of the antiviral role of RH30 helicase illustrates the likely ancient roles of RNA helicases in plant innate immunity. In summary, we have demonstrated that the plant DDX17-like RH30 DEAD-box helicase acts as a major restriction factor against tombusvirus replication when expressed in plants and yeast surrogate host. We show that RH30 DEAD-box helicase is targeted to the large TBSV replication compartment. In addition, we find that RH30 blocks the assembly of viral replicase complex, the activation of the RNA-dependent RNA polymerase function of p92^pol^ and binding of p33 replication protein to critical *cis*-acting element in the TBSV RNA (Fig 12). Altogether, the plant DDX17-like RH30 DEAD-box helicase is a potent, effector-type, restriction factor of tombus- and related viruses.

## Materials and methods

### Biotinylated RNA-protein interaction assay

Biotinylated RII RNA of DI-72(+) was synthesized by *in vitro* T7 transcription in the presence of 7.5 μl of 10 mM ATP, CTP, GTP and 5 mM UTP as well as 0.35 μl of 10 mM biotin16-UTP (Roche) in a total of 50 μl reaction volume. The interaction assay was performed with 3.8 μM of recombinant MBP-RH30 and 1.9 μM of MBP-p33C along with 0.1 μg of biotinylated RNA, 0.1 μl of tRNA (1 mg/ml), 2 U RNase inhibitor, and 1 mM ATP in the presence of biotin-RNA binding buffer (100 mM Tris [pH 7.9], 10% glycerol, 100 mM KCl, 5 mM MgCl_2_, 0.1% NP-40) in a 10 μl reaction mixture. Non-biotinylated RII of DI-72(+) RNA or absence of ATP was used as controls.

Assay #1: Recombinant MBP-RH30 was incubated first with biontinylated RII(+) RNA at 25°C for 15 min. Then, the recombinant MBP-p33C was added to the reaction and incubated for another 15 min. Assay #2: Recombinant MBP-RH30 and MBP-p33C were co-incubated simultaneously with biontinylated RII(+) RNA at 25°C for 30 min. The reaction mixtures were incubated with 20 μl of Promega Streptavidin MagneSphere Paramagnetic Particles (VWR) at room temperature for 20 min. The particles were collected in a magnetic stand and washed with binding buffer for five times. The protein-RNA complexes were then eluted with 20 μl of SDS loading dye containing β-mercaptoethanol by boiling for 15 min. The eluted samples were analyzed by Western blot with anti-p33 antibody.

Assay #3: For the detection of p33 released from protein-biotinylated RNA complex, 1.9 μM of recombinant MBP-p33C was incubated with 0.1 μg of biontinylated RII of DI-72(+) RNA at 25°C for 15 min, followed by the addition of 20 μl of Promega Streptavidin MagneSphere Paramagnetic Particles for another 30 min incubation at room temperature. After collection of the beads and washing with biotin-RNA binding buffer for five times, the particles were incubated with either 0.95 or 3.8 μM of MBP-RH30 or MBP (used as control) in the presence of biotin-RNA binding buffer containing 1 mM ATP at 25°C for 15 min. The supernatant of the mixture was collected after collecting the particles in a magnetic stand and was analyzed by Western blot with anti-p33 antibody.

### Gel mobility shift assay (EMSA) and dsRNA strand-separation assay

The conditions for the EMSA experiments were described previously [24]. Briefly, the EMSA assay was performed with 0.1 pmol of ^32^P-labeled RNA probes along with different concentrations (0.4, 1.9, and 5.7 μM) of purified recombinant MBP-fusion proteins or MBP in the presence of RNA binding buffer (10 mM HEPES [pH7.4], 50 mM NaCl, 1 mM DTT, 1 mM EDTA, 5% Glycerol, 2.5 mM MgCl_2_), 2 U of RNase inhibitor, as well as 0.1 μg of tRNA in a total of 10 μl reaction volume. Two different amounts (2 and 4 pmol) of unlabeled RNAs together with 5.7 μM of either MBP-RH30 or MBP were used for template competition.

To study if purified proteins could unwind partial dsRNA duplex, the dsRNA strand-separation assay was performed as described [28]. Firstly, the unlabeled single-stranded DI-72 (-) or DI-72 (+) RNAs were synthesized via T7 polymerase- based *in vitro* transcription. The ^32^P-labeled single-stranded RI(-) or RII(+) RNAs were synthesized by T7-based *in vitro* transcription using ^32^P-labeled UTP. To prepare partial dsRNA duplexes, consisting of either RI(-)/DI-72 (+) or RII(+)/DI-72 (-) (see Fig 7E and 7F), 2 pmol of ^32^P -labeled RI(-) or RII(+) were annealed to 6 pmol of unlabeled DI-72(+) or DI-72 (-) in STE buffer (10 mM TRIS [pH 8.0], 1 mM EDTA, and 100 mM NaCl) by slowly cooling down the samples (in a total volume of 20 μl) from 94°C to 25°C in 30 min. To test if the purified recombinant proteins could separate the partial dsRNA duplex, 1.9 and 5.7 μM purified MBP fusion proteins or MBP as a negative control were added separately to the partial dsRNA duplex in the RNA binding buffer (10 mM HEPES [pH7.4], 50 mM NaCl, 1 mM DTT, 1 mM EDTA, 5% Glycerol, 2.5 mM MgCl_2_) along with 1mM ATP, followed by incubation at 25°C for 25 min. The reaction mixtures were then treated with Proteinase K (2 μg/per reaction) at 37°C for 20 min, followed by loading onto 5% nondenaturing polyacrylamide gel with 200V for 1 h. Additional methods can be found in S1 Text and the primers used are listed in S1 Table.

## Supporting information

S1 Text Materials and methods. (DOCX)

S1 Table Sequences of primers used in this study. (DOCX)

S1 Fig Comparison of the conserved F position in the helicase core domain in the yeast Ded1 and the Arabidopsis RH30 DEAD-box helicases. (DOCX)

S2 Fig Expression of AtRH30 DEAD-box helicase inhibits NoV RNA accumulation in yeast. (A-B) Top panel: Northern blot analysis of NoV (panel A) or FHV (panel B) RNA1 and the subgenomic RNA3 using a 3’ end specific probe shows the reduced accumulation of NoV but not FHV RNAs in WT yeast strain expressing His_6_-RH30. Viral replication proteins, termed protA, were expressed with a Flag-tag from plasmids from the *GAL1* promoter, while the viral RNA templates were also expressed from the *GAL1* promoter. His_6_-RH30 was expressed from a plasmid. Middle panel: Northern blot with 18S ribosomal RNA specific probe was used as a loading control. Bottom images: Western blot analyses of the level of Flag-protA with anti-Flag antibody and His_6_-RH30 with anti-His antibody are shown in the bottom panels with total protein extracts from yeast stained with Coomassie Blue.
(EPS)
