# Supplementary material for: Blocking tombusvirus replication through the antiviral functions of DDX17-like RH30 DEAD-box helicase
Source: PLoS Pathog. 2019 May 28;15(5):e1007771. doi: 10.1371/journal.ppat.1007771 (PMC6555533; doi:10.1371/journal.ppat.1007771)
Supplement: S1 Text — (DOCX) [file ppat.1007771.s001.docx]

**S1 text. Materials and methods**

**Yeast strains and expression plasmids.** The yeast (*Saccharomyces cerevisiae*) strains BY4741 (MATa his3Δ1 leu2Δ0 met15Δ0 ura3Δ0), and TET::DED1 (yTHC library) were obtained from Open Biosystems. RT-PCR products of *Arabidopsis RH30* gene and its mutant RH30^F416L^ were obtained as follows: Total RNA from *Arabidopsis* was isolated and used for RT-PCR with primers #5753 and #5754 to obtain the sequence of *RH30*. Meanwhile, two PCR-generated fragments that partly overlap with each other and introduce the point mutation in RH30 were amplified with RT-PCR using primers #6706 and #5753 or #5754 and #6707. These two PCR-generated fragments were then used as templates to obtain the whole sequence of RH30^F416L^ by PCR. To generate plasmids for expression of *Arabidopsis RH30* and RH30^F416L^ in yeast and plants, the obtained PCR products were digested with XhoI and XbaI and then inserted into XhoI/XbaI digested pYES-NT and pGD-35S, resulting in pYES-RH30, pGD-RH30, and pGD-RH30^F416L^.

To prepare expression plasmids for recombinant protein purification from E.*coli*, Arabidopsis RH30 and RH30^F416L^ sequences were PCR-amplified with primers #6061 and #6062 using pGD-RH30 and pGD-RH30^F416L^ plasmids, respectively. The obtained PCR products were digested with XbaI and XhoI, followed by the ligation into XbaI/SalI digested pMAL-c2x, generating pMAL-RH30 and pMAL-RH30^F416L^.

To obtain plasmids for expression of N-terminal Green Fluorescent Protein (GFP) or Red Fluorescent Protein (RFP)-tagged proteins, Arabidopsis RH30 sequence was RT-PCR-amplified with primers #5754 and #6839 and digested with XhoI and ApaI, followed by ligation into XhoI/ApaI digested pGDG and pGDR [1], respectively, resulting in pGD-GFP-RH30 and pGD-RFP-RH30.

The sequence of bacteriophage MS2 coat protein gene was PCR-amplified from pGBK-MS2CP-EYFP [2] with primers #1567 and #1568, followed by digestion with XhoI and BamHI. The PCR product of mRFP was obtained by PCR-based amplification with primers #2691 and # 5051, and the obtained PCR product was digested with BglII and XbaI. These two digested PCR products were co-inserted into XhoI/XbaI digested pGD-35S to generate pGD-MS2CP-RFP. The sequence of the full-length DI-72 carrying of six repeats of MS2 hairpin [2], which binds specifically to the MS2 phage coat protein, [3] and a 3’ ribozyme were PCR-amplified from pYC-DI-72(+)-MS2 or pYC-DI-72(-)-MS2 [2] with primers #471 and #1069. The obtained PCR products were digested with XhoI and SacI, followed by ligation into XhoI/SacI digested pGD-35S, creating pGD-DI-72(+)-MS2hp and pGD-DI-72(-)-MS2hp.

To make *Arabidopsis* RH30 restricted in localization to the nucleus, a nuclear retention signal (NRS) was fused to RH30. The NRS fragment was PCR-amplified from pCiNeo-3XFlag-NRS-NCL [4] (a generous gift from Dr. Glaunsinger) using primers #6877 and #6876, followed by digestion with XhoI and HindIII. RH30 sequence was PCR-amplified from pGD-RH30 using primers #6880 and #5753, followed by digestion with HindIII and XbaI. These two digested fragments were then inserted to XhoI/XbaI digested pGD-35S, generating pGD-His6-NRS-RH30. GFP sequence was PCR-amplified from pGDG using primers #6512 and #6513. This PCR product was then digested with XhoI and SacI, followed by the ligation into SalI/SacI digested pGD-His6-NRS-RH30. This created a plasmid pGD-His6-NRS-RH30-GFP, which expresses C-terminal GFP-tagged NRS-RH30.

To generate the expression plasmids for the BiFC assays in plants, the sequence of *Arabidopsis* RH30 was PCR-amplified from pGD-RH30 with primers #5754 and #5753, followed by the digestion with XbaI and XhoI. Also, the N-terminal half of yellow fluorescence protein (nYFP) sequence was PCR-amplified using pGD-nYFP-MBP plasmid as template [5] and primers #5905 and #6069, followed by the digestion with BglII and BamHI. BglII/BamHI digested nYFP fragment was ligated into BamHI digested pGD-35S, resulting in pGD-nYFP. The pGD-nYFP plasmid was then digested with XbaI and XhoI and was used for the ligation with XbaI/XhoI digested RH30, generating pGD-nYFP-RH30.

**Tombusvirus replication assay in yeast.** To test tombusvirus replication in yeast, BY4741 strain of *Saccharomyces cerevisiae* was transformed with LpGAD-CUP1::HisFlag-p92 and HpGBK-CUP1::HisFlag-p33/GAL1::DI-72 together with pYES-empty (as control), pYES-RH30 or pYES-RH30^F416L^. The obtained yeast transformants were grown in SC-ULH^-^ media containing 2 % galactose and 0.1 mM BCS at 29 °C. After 18h, the yeast culture was transferred to SC-ULH^-^ media supplemented with 2% galactose and 50 µM CuSO_4_ and incubated at 23°C for 7h. The obtained yeast cells were used for further Northern blot analysis and Western blot analysis [2].

To test FHV replication in yeast, BY4741 strain was transformed with HpESC-Gal 1::FHV RNA1-frameshift and LpGAD-CUP1::cHaFlag-FHV protein A [6] along with pYES-empty (as control), pYES-RH30 or pYES-RH30^F416L^. The transformed yeast cells were grown in SC-ULH^-^ media supplemented with 2 % galactose and 0.1 mM BCS at 23 °C for 18 h. After that, the yeast cultures were transferred to SC-ULH^-^ media supplemented with 2% galactose and 50 µM CuSO_4_ and incubated at 23°C for 48 h. The yeast cells were collected for further Northern blot analysis and Western blot analysis.

To test NoV replication in yeast, BY4741 strain was transformed with HpESC-CUP1::NOV RNA1-frameshift and LpESC-CUP1:: cHaFlag-NOV protein A [6] along with pYES-empty (as control), pYES-RH30 or pYES-RH30^F416L^. The obtained yeast transformants were grown in SC-ULH^-^ media supplemented with 2 % galactose and 0.1 mM BCS at 29 °C for 18 h. The yeast culture was then transferred to SC-ULH^-^ media containing 2 % galactose and 50 µM CuSO_4_ and incubated at 29 °C for 48 h.

**Recombinant protein purification from E. coli.** Recombinant proteins MBP-RH30, MBP-RH30^F416L^, MBP-p33, MBP-p92 and MBP were expressed in E. *coli* and affinity-purified as described [7]. Briefly, E. *coli* strain BL21 (DE3) CodonPlus (Stratagene) cells were transformed with the above plasmids to express the recombinant proteins. Then, the E. *coli* cells were cultured at 37°C for 16h, followed by dilution of the culture to OD_600_ 0.2 with fresh media. The E. *coli* cultures were incubated at 37°C until reaching 1.0 OD_600_. Subsequently, the E. *coli* cultures were incubated at 16°C in the presence of isopropyl-β-D-thiogalactopyranoside (IPTG) for 8 h. The E. *coli* cells were then collected by centrifugation at 5000 rpm for 5 min at 4°C, followed by the resuspension in ice-cold column buffer (20mM HEPES [pH7.4], 25 mM NaCl, 1mM EDTA [pH 8.0]) containing 10 mM β-mercaptoethanol and 1 µl of RNase A (1 mg/ml) per 4 ml of E. *coli* cell-suspension. The cells were then sonicated on ice and the lysates were centrifuged at 15,000 rpm for 15 min at 4°C. The obtained supernatants were incubated with amylose resin (NEB) at 4°C for 2 h. After the resin was washed with column buffer, the recombinant proteins were eluted with column buffer containing 0.36% [W/V] maltose and 1mM DTT.

**Co-purification of RH30 with the Tombusvirus replication complex.** Yeast BY4741 strain was transformed with plasmids pYES-RH30, HpGBK-CUP1::FLAGp33/GAL1::DI-72, and LpGAD-CUP1::FLAGp92, while yeasts transformed with pYES-RH30, HpGBK-CUP1::Hisp33/GAL1::DI-72 and LpGAD-CUP1::Hisp92 were used as control. The assay was performed as described [8,9] with minor modification. Briefly, the obtained yeast transformants were grown in SC-ULH^-^ media containing 2% glucose at 23°C for 16 h. The culture was then transfer to SC-ULH^-^ media supplemented with 2% galactose for another 24 h at 23°C, followed by the addition of 50 µM CuSO_4_ and incubation for 6 h at 23°C. The obtained yeast cells were resuspended in high salt TG buffer (50 mM Tris-HCl [pH 7.5], 10% glycerol, 0.5 M NaCl, 10 mM KCl, 15 mM MgCl_2,_ 1% [V/V] yeast protease inhibitor cocktail [Ypic]) and broken in a FastPrep Homogenizer (MP Biomedicals) with glass beads, followed by centrifugation at 500 g for 5 min at 4°C. The membrane fraction containing viral replicase complex was collected by centrifugation at 35,000 g for 20 min at 4°C, followed by solubilization in high salt TG buffer containing 2 % Triton X-100, 1% [V/V] Ypic for 3 h at 4°C. The supernatant of detergent-solubilized membranes was collected by centrifugation at 35,000 g for 20 min at 4°C and then was incubated with anti-FLAG M2-agarose affinity resin (Sigma) in columns for 16 h at 4°C. After that, the columns were washed with high salt buffer for three times. To elute the protein samples from the column, the preparations were incubated with SDS-PAGE loading dye at 85°C for 6 min, followed by centrifugation at 500xg for 3 min. β-mercaptoethanol was added to the samples, then they were boiled for 20 min. Affinity-purified p33 was analyzed by Western blot with anti-FLAG antibody, and co-purified 6xHis-tagged RH30 was analyzed by Western blot with anti-His antibody.

**Pull-down assay.** E. *coli* strain BL21 (DE3) CodonPlus (Stratagene) cells were transformed with expression plasmids for expression and purification of recombinant proteins, including MBP-RH30, MBP-RH30^F416L^, GST-TBSV p33C and MBP. The methods to obtain E. *coli* cell lysate as described previously [10,11]. E. *coli* lysates containing MBP, MBP-AtRH30, or MBP-RH30^F416L^ were separately incubated with amylose resin (NEB) for 2 h at 4°C, followed by washing with cold column buffer three times. The amylose resin was then incubated with E. *coli* lysates containing GST-TBSV p33C for 4 h at 4 °C in the presence of 0.5 % NP-40 and 0.1 % [V/V] Ypic, followed by washing with cold column buffer containing 0.5 % NP-40 three times. The protein complexes bound to resin were eluted with cold column buffer containing 0.36% [W/V] maltose and 1mM DTT, followed by the analysis with Western blot assay.

**Yeast cell-free extract (CFE)-based *in vitro* TBSV replication assay and *in vitro* RdRp activation assay.** The yeast CFE that supports TBSV RNA replication in vitro was prepared using BY4741 yeast strain as described [12,13]. The *in vitro* CFE assay #1 (Fig. 6) was performed with the mixture of 2 µl of CFE, 0.5 µg DI-72 (+)repRNA, 0.2 µg affinity-purified maltose-binding protein (MBP)-p33 as well as MBP-p92^pol^ (both recombinant proteins were purified from E. *coli*) [10], 5 µl of buffer A (30 mM HEPES-KOH [pH 7.4], 150 mM potassium acetate, 5 mM magnesium acetate, 0.13 M sorbitol), 2 µl of 150 mM creatine phosphate, 0.2 µl of 10 mg/ml creatine kinase, 0.4 µl actinomycin D (5mg/ml), 0.2 µl of 1 M dithiothreitol (DTT), 0.2 µl of RNase inhibitor, 2 µl a ribonucleotide (rNTP) mixture (10 mM of ATP, CTP, and GTP as well as 0.25 mM UTP), 0.1 µl of [^32^P]UTP and affinity-purified recombinant proteins MBP-RH30, MBP-RH30^F416L^, or MBP in a total of 20 µl reaction volume. The reaction was performed at 25°C for 3h and then stopped by the addition of a 110 µl of 1% SDS and 50 mM EDTA, followed by phenol-chloroform extraction and RNA precipitation. In order to detect the amount of dsRNA, the obtained ^32^P-labeled repRNA products were then divided into two halves: one was heat denatured at 85°C for 5 min in the presence of 50% formamide, while the other one was not denatured. Then, the repRNA products were analyzed by electrophoresis in a 5% polyacrylamide gel (PAGE) containing 8 M urea and 0.5X Tris-borate-EDTA (TBE) buffer.

To dissect the mechanisms of RH30 antiviral function in tombusvirus replication, a step-wise *in vitro* CFE replication assay #2 (Fig. 6) was performed. The purified proteins (MBP or MBP-RH30) were added during either step 1 reaction (i.e., VRC assembly step) or step 2 reaction (i.e., tombusviral RNA synthesis step) [12]. In the first step, a mixture of 2 µl of yeast CFE, 0.5 µg DI-72 (+)repRNA transcripts, 0.2 µg MBP-p33 and MBP-p92^pol^, 5 µl of buffer A, 2 µl of 150 mM creatine phosphate, 0.2 µl of 10 mg/ml creatine kinase, 0.4 µl actinomycin D (5mg/ml), 0.2 µl of 1 M DTT, 0.2 µl of RNase inhibitor, 2 µl of 10 mM ATG and GTP mixture in a 20 µl reaction volume, followed by incubation at 25°C for 1 h. The reaction mixture was centrifuged at 15,000 rpm for 10 min at 4°C. The collected membrane-fraction of CFE, which contains the membrane-bound VRCs, was washed with 100 µl of buffer A for once, followed by centrifugation at 15,000 rpm for 10 min at 4°C. The obtained membrane preparations were dissolved in 8 µl buffer A. In second step, the 8 µl of collected samples was added to 12 µl reaction mixture composed of 3 µl of buffer A, 2 µl of 150 mM creatine phosphate, 0.2 µl of 10 mg/ml creatine kinase, 0.4 µl actinomycin D (5mg/ml), 0.2 µl of 1 M DTT, 0.2 µl of RNase inhibitor, 2 µl of rNTP mixture (10 mM of ATP, CTP, and GTP as well as 0.25 mM UTP) and 0.1 µl of [^32^P]UTP [12]. Then, the assays were performed at 25°C for 3 h, and stopped by the addition of a 1/10 volume of 1% SDS and 50 mM EDTA, followed by phenol-chloroform extraction and RNA precipitation [12].

For the *in vitro* RdRp activation assay, the CFE soluble fraction and recombinant affinity purified MBP-p92-Δ167N was used as described [14]. The CFE soluble fraction (supernatant) was collected by centrifugation of the original CFE at 42,000 g for 20 min at 4°C [12,13]. Then 2 µl of the obtained CFE soluble fraction and approximately 0.2 µg of MBP-p92-Δ167N along with different concentration (1.9, and 3.8 µM) of MBP-RH30 or MBP were incubated with 5 µl of buffer A (30mM HEPES-KOH [pH 7.4], 150 mM potassium acetate, 5 mM magnesium acetate, 0.13 M sorbitol), 2 µl of 150 mM creatine phosphate, 0.2 µl of 10 mg/ml creatine kinase, 0.4 µl actinomycin D (5 mg/ml), 0.2 µl of 1 M dithiothreitol (DTT), 0.2 µl of RNase inhibitor, 2 µl a ribonucleotide (rNTP) mixture (10 mM of ATP, CTP, and GTP as well as 0.25 mM UTP) and 0.1 µl of [^32^P]UTP in a total of 20 µl reaction volume. The following reaction was then performed and analyzed as described [15].

**In vitro translation assay.** To test if AtRH30 influences the translation of tombusvirus genomic RNA, an *in vitro* translation assay was performed as described [16]. Briefly, approximately 0.5 µg of CIRV genomic RNA and TDH2 mRNA were incubated with different concentrations (1.9 µM, and 3.8 µM) of recombinant MBP-RH30 along with 2.5 µl of wheat germ extract, 0.4 µl amino acid mix (minus Methionine), 0.47 µl of 1M KOAC, 6 U of RNase inhibitor, 0.1 µl of [^35^S]Methionine in a total of 10 µl reaction volume. After 1.5 h incubation at room temperature, the samples were boiled with SDS-PAGE loading dye for 5 min, followed by analysis with 10 % acrylamide gel.

**Confocal microscopy.** The subcellular localization of *Arabidopsis* RH30 in plant epidermal cells or protoplasts was observed with the help of N-terminal fusion of RH30 to GFP. Protoplasts were isolated from *N. benthamiana* leaves as described [17,18]. The wild-type or transgenic N. benthamiana (constitutively expressing H2B fused to RFP) leaves were infiltrated with agrobacterium carrying expression plasmids pGD-GFP-RH30 (OD_600_ 0.3), pGD-p33-BFP (OD_600_ 0.3), pGD-P19 (OD_600_ 0.2), pGD-CNV^20KSTOP^ (OD_600_ 0.2). The wild-type *N. benthamiana* leaves were co-infiltrated with agrobacterium carrying pGD-RFP-SKL (OD_600_ 0.3) to visualize peroxisomes. The absence of pGD-CNV^20KSTOP^ or pGD-p33-BFP was used as control. Approximately 72 h post-agroinfiltration, imaging of infiltrated leaves or protoplasts was performed on an Olympus FV1200 confocal microscopy using 40X or 60X water-immersion objective equipped lasers. BFP was excited with 405 nm laser, GFP was excited with 488 nm laser, and RFP was excited with 543 nm laser. Images were obtained and merged using Olympus FLUOVIEW 1.5.

The subcellular localization of repRNA(+)-MS2hp and repRNA(-)-MS2hp RNAs was observed in plant epidermal cells with C-terminal fusion of MS2 coat protein to RFP, which recognizes MS2 six hairpins inserted into repRNA(+) and repRNA(-) [2]. The N. benthamiana leaves were infiltrated with agrobaterium carrying pGD-CNV^20KSTOP^, pGD-P19, pGD-p33-BFP, pGD-GFP-RH30, and pGD-DI-72(+)-MS2hp or pGD-DI-72(-)-MS2hp (OD_600_ 0.2 of each). The absence of pGD-GFP-RH30, pGD-DI-72(+)/(-)-MS2hp or pGD-CNV^20KSTOP^ was used as control. Approximately 84 h post-infiltration, imaging of infiltrated leaves was obtained as described above.

To visualize the subcellular localization of tombusvirus dsRNA, *N. benthamiana* leaves were co-infiltrated with pGD-CNV^20KSTOP^ (OD_600_ 0.2), pGD-P19 (OD_600_ 0.2), pGD-p33-BFP (OD_600_ 0.2), pGD-RFP-RH30 (OD_600_ 0.2), pGD-VP35-YC (OD_600_ 0.1), and pGD-B2-YN (OD_600_ 0.1) (a generous gift from Dr. Aiming Wang) [19]. The absence of pGD-RFP-RH30, pGD-CNV^20KSTOP^, or pGD-p33-BFP was used as control. Approximately 84 h post-infiltration, imaging of infiltrated leaves was obtained as described above except YFP was excited with 488 nm laser.

**Bimolecular fluorescence complementation assay.** The interaction between TBSV p33 replication protein and AtRH30 helicase was detected in *N. benthamiana* leaves by bimolecular fluorescence complementation (BiFC). The *N. benthamiana* leaves were infiltrated with agrobacterium carrying pGD-p19 (OD_600_ 0.2), pGD-RFP-SKL (OD_600_ 0.4), pGD-T33-cYFP (OD_600_ 0.4) [5] and pGD-nYFP-RH30 (OD_600_ 0.4) or pGD-nYFP-MBP (as a control, OD_600_ 0.4), followed by inoculation with TBSV crude sap inoculum 16 h after agro-infiltration. Two days post-virus inoculation, confocal microscopy imaging of infiltrated leaves was performed as described above.

**Virus accumulation in *N. benthamiana* expressing AtRH30 and in RH30 knockout Arabidopsis plants.** *N. benthamiana* leaves were co-infiltrated with agrobacterium carrying pGD-RH30 and pGD-P19. In the experiment of CNV^20KSTOP^ or TMV infection, plants were also co-infiltrated with agrobacterium carrying pGD-CNV^20KSTOP^ or pJL-36 for TMV [20]. In the experiment for TBSV, CIRV, TCV, and RCNMV infections, plants were inoculated with crude sap inocula 16 h after agro-infiltration. About 36 h (for TBSV infection); 48 h (for CNV, CIRV and TMV infections); 72 h (for RCNMV); 144 h (for TCV) post-virus inoculation, the virus-inoculated leaves were collected for total RNA extraction and Northern blot as described [21] to analyze the accumulation levels of these viruses.

To detect the accumulation level of TCV in RH30 knockout *Arabidopsis* plants, transgenic *Arabidopsis* line (#CS372806) containing T-DNA insertion within endogenous RH30 ORF was obtained from the Arabidopsis Biological Resource Center. After self-fertilization and confirmation by genotyping, the homozygous lines were collected and the leaves were inoculated with TCV crude sap inoculum. After 48h post-inoculation, total RNA from inoculated leaves was extracted and analyzed by Northern blot as described above.

**VIGS-based knockdown of RH30 in *N. benthamiana* plants.** To knockdown the expression levels of endogenous NbRH30 in *N. benthamiana* plants, virus-induced gene silencing (VIGS) assay was performed as described [22,23]. The predicted cDNA sequence of NbRH30 (Accession number: Nbv5.1tr6207343) was obtained by a blast search using the sequence of AtRH30 in QUT *Nicotiana benthamiana* database. To generate the VIGS vectors (pTRV2-Nb30-5, targeting 5’ region in NbRH30 mRNA; pTRV2-Nb30-3, targeting 3’ region in NbRH30 mRNA), an NbRH30 gene fragment was PCR-amplified from *N. benthamiana* cDNA using primers #7304 and #7307. This fragment was used as a template to obtain two 300-bp cDNA fragments encoding 5’ or 3’ region of NbRH30 gene via PCR using primers #7304 and #7305 or #7306 and #7307, respectively. The obtained fragments were digested with BglII and SalI, respectively, followed by the ligation into BamHI/XhoI digested pTRV2-empty, resulting in pTRV2-Nb30-5 or pTRV2-Nb30-3.

The leaves of *N. benthamiana* plants were infiltrated with agrobacterium carrying pTRV1 together with pTRV2-Nb30-5 (targeting 5’ region in NbRH30 mRNA) or pTRV2-Nb30-3 (targeting 3’ region in NbRH30 mRNA) or pTRV2-cGFP (as a control). 12 days post-infiltration, the RH30 mRNA level in upper systemic leaves were investigated by semi-quantitative RT-PCR with primers #7306 and #7307 (in case of TRV1/TRV2-Nb30-5 silenced plants); primers #7304 and #7305 (in case of TRV1/TRV2-Nb30-3 silenced plants). The levels of 18S rRNA or tubulin mRNA were used as internal control in Northern blotting or RT-PCR using primers #2859 and #2860. After the silencing effects were confirmed, the silenced upper leaves were inoculated with TBSV crude sap. Approximately 36 h post-inoculation, the total RNA of inoculated leaves were extracted and analyzed by Northern blot as described above.

The expression level of NbRH30 mRNA in upper systemic leaves was investigated by Northern blotting assay. The ^32^P-labeled probes targeting either 5’ or 3’-regions of NbRH30 mRNA for the detection in Northern blotting were prepared as follows: The 5’ or 3’-regions of NbRH30 sequence were PCR-amplified with primers #7304 and #7990 or #7306 and #7991, respectively. The obtained PCR-products were utilized as templates for *in vitro* T7-trancription along with [^32^P]UTP to produce ^32^P-labeled probes. The probe targeting 5’-region of NbRH30 mRNA was used for plants silenced by TRV1/TRV2-Nb30-3, while the probe targeting 3’-region of NbRH30 mRNA was used for plants silenced by TRV1/TRV2-Nb30-5.

For the detection of TMV genomic RNA in plants, we utilized a ^32^P-labeled probe targeting 3’-region of TMV gRNA. The probe was prepared by PCR-amplification of the 3’ proximal of TMV genome with primers #6192 and #6193 from plasmid pJL-36. The obtained PCR products were then used as templates for *in vitro* T7-trancription along with [^32^P]UTP to generate the ^32^P-labeled probe for Northern blotting assay.

**References**

1. Goodin MM, Dietzgen RG, Schichnes D, Ruzin S, Jackson AO (2002) pGD vectors: versatile tools for the expression of green and red fluorescent protein fusions in agroinfiltrated plant leaves. Plant J 31: 375-383.

2. Panavas T, Hawkins CM, Panaviene Z, Nagy PD (2005) The role of the p33:p33/p92 interaction domain in RNA replication and intracellular localization of p33 and p92 proteins of Cucumber necrosis tombusvirus. Virology 338: 81-95.

3. Bertrand E, Chartrand P, Schaefer M, Shenoy SM, Singer RH, et al. (1998) Localization of ASH1 mRNA particles in living yeast. Mol Cell 2: 437-445.

4. Muller M, Hutin S, Marigold O, Li KH, Burlingame A, et al. (2015) A ribonucleoprotein complex protects the interleukin-6 mRNA from degradation by distinct herpesviral endonucleases. PLoS Pathog 11: e1004899.

5. Xu K, Nagy PD (2016) Enrichment of Phosphatidylethanolamine in Viral Replication Compartments via Co-opting the Endosomal Rab5 Small GTPase by a Positive-Strand RNA Virus. PLoS Biol 14: e2000128.

6. Pogany J, Panavas, T., Serviene, E., Nawaz-Ul-Rehman, MS., and Nagy, PD (2010) A high-throughput approach for studying virus replication in yeast. Current Protocols in Microbiology 19: 16J.11.11-16J.11.15.

7. Kovalev N, Pogany J, Nagy PD (2012) A Co-Opted DEAD-Box RNA Helicase Enhances Tombusvirus Plus-Strand Synthesis. PLoS Pathog 8: e1002537.

8. Panaviene Z, Panavas T, Serva S, Nagy PD (2004) Purification of the cucumber necrosis virus replicase from yeast cells: role of coexpressed viral RNA in stimulation of replicase activity. J Virol 78: 8254-8263.

9. Barajas D, Martin IF, Pogany J, Risco C, Nagy PD (2014) Noncanonical Role for the Host Vps4 AAA+ ATPase ESCRT Protein in the Formation of Tomato Bushy Stunt Virus Replicase. PLoS Pathog 10: e1004087.

10. Rajendran KS, Nagy PD (2003) Characterization of the RNA-binding domains in the replicase proteins of tomato bushy stunt virus. J Virol 77: 9244-9258.

11. Rajendran KS, Pogany J, Nagy PD (2002) Comparison of turnip crinkle virus RNA-dependent RNA polymerase preparations expressed in Escherichia coli or derived from infected plants. J Virol 76: 1707-1717.

12. Pogany J, Stork J, Li Z, Nagy PD (2008) In vitro assembly of the Tomato bushy stunt virus replicase requires the host Heat shock protein 70. Proc Natl Acad Sci U S A 105: 19956-19961.

13. Pogany J, Nagy PD (2008) Authentic replication and recombination of Tomato bushy stunt virus RNA in a cell-free extract from yeast. J Virol 82: 5967-5980.

14. Pogany J, Nagy PD (2015) Activation of Tomato Bushy Stunt Virus RNA-Dependent RNA Polymerase by Cellular Heat Shock Protein 70 Is Enhanced by Phospholipids In Vitro. J Virol 89: 5714-5723.

15. Pogany J, Nagy PD (2012) p33-Independent Activation of a Truncated p92 RNA-Dependent RNA Polymerase of Tomato Bushy Stunt Virus in Yeast Cell-Free Extract. J Virol 86: 12025-12038.

16. Cimino PA, Nicholson BL, Wu B, Xu W, White KA (2011) Multifaceted regulation of translational readthrough by RNA replication elements in a tombusvirus. PLoS Pathog 7: e1002423.

17. Panaviene Z, Baker JM, Nagy PD (2003) The overlapping RNA-binding domains of p33 and p92 replicase proteins are essential for tombusvirus replication. Virology 308: 191-205.

18. Xu K, Nagy PD (2015) RNA virus replication depends on enrichment of phosphatidylethanolamine at replication sites in subcellular membranes. Proc Natl Acad Sci U S A 112: E1782-E1791.

19. Cheng X, Deng P, Cui H, Wang A (2015) Visualizing double-stranded RNA distribution and dynamics in living cells by dsRNA binding-dependent fluorescence complementation. Virology 485: 439-451.

20. Lindbo JA (2007) High-efficiency protein expression in plants from agroinfection-compatible Tobacco mosaic virus expression vectors. BMC Biotechnol 7: 52.

21. Kovalev N, Nagy PD (2014) The Expanding Functions of Cellular Helicases: The Tombusvirus RNA Replication Enhancer Co-opts the Plant eIF4AIII-Like AtRH2 and the DDX5-Like AtRH5 DEAD-Box RNA Helicases to Promote Viral Asymmetric RNA Replication. PLoS Pathog 10: e1004051.

22. Dinesh-Kumar SP, Anandalakshmi R, Marathe R, Schiff M, Liu Y (2003) Virus-induced gene silencing. Methods Mol Biol 236: 287-294.

23. Jaag HM, Nagy PD (2009) Silencing of Nicotiana benthamiana Xrn4p exoribonuclease promotes tombusvirus RNA accumulation and recombination. Virology 386: 344-352.
