# Supplementary material for: Blocking tombusvirus replication through the antiviral functions of DDX17-like RH30 DEAD-box helicase
Source: PLoS Pathog. 2019 May 28;15(5):e1007771. doi: 10.1371/journal.ppat.1007771 (PMC6555533; doi:10.1371/journal.ppat.1007771)
Supplement: S1 Table — (DOCX) [file ppat.1007771.s002.docx]

**S1 Table. Sequences of primers used in this study.**

| Primers (# NO.) | Sequences |
| --- | --- |
| 471 | CCCGCTCGAGGGAAATTCTCCAGGATTTCTC |
| 1069 | CCGGTCGAGCTCTACCAGGTAATATACCACAACGTGTGT |
| 1567 | GCAGCTCGAGACCATGCCAAAAAAGAAAAGAAAAAGTGGCT |
| 1568 | CGACGGATCCGTAGATGCCGGAGTTT |
| 2691 | CGGAGATCTATGGCCTCCTCCGAGGAC |
| 2859 | TAATACGACTCACTATAGGAACCAAATCATTCATGTTGCTCTC |
| 2860 | TAGTGTATGTGATATCCCACCAA |
| 5051 | GGATCTAGATTAGGCGCCGGTGGAGTGG |
| 5753 | CGCGTCTAGATTACCAAGTCCTCTTTCCAC |
| 5754 | CGCGCTCGAGATGAGCTCGTATGATCGTAG |
| 5905 | GGAAGATCTATGGTGAGCAAGGGCGAG |
| 6061 | CGCGTCTAGAATGAGCTCGTATGATCGTAG |
| 6062 | CGCGCTCGAGTTACCAAGTCCTCTTTCCAC |
| 6069 | GCGCGGATCCGTCCTCGATGTTGTGGC |
| 6512 | CCGCTCGAGCTGAGTCCGGACTTGTATAG |
| 6192 | CGCAACAAGCTAGGACAACAGTCC |
| 6193 | TAATACGACTCACTATAGGGCCGCTACCGGCGGTTAGGGGAGG |
| 6513 | CGCGGAGCTCTTACTGAGTCCGGACTTGTATAG |
| 6706 | ATGGGAGTAAAATCCTAATTTTGGTGGAGACAAAGAGAGGGTGTG |
| 6707 | CACACCCTCTCTTTGTCTCCACCAAAATTAGGATTTTACTCCCAT |
| 6839 | CCGGGCCCTTACCAAGTCCTCTTTCCAC |
| 6876 | CCCAAGCTTGCCAACTTTTTTGTACAAAC |
| 6877 | CCGCTCGAGATGCATCATCACCATCACCATATGCCAAAAGTGAACCGAGGAA |
| 6880 | ATGAGCTCGTATGATCGTAG |
| 7304 | GAAGATCTATGAGCTACTCTAATTACGACTCC |
| 7305 | ACGCGTCGACAAAGCCACTGTCCCGGCCCATAC |
| 7306 | GAAGATCTTATGATTTCCCTTCAAATCTTGAGG |
| 7307 | ACGCGTCGACCTACCAAGGCCTTCTACCAAGC |
| 7990 | GGAGTTAATACGACTCACTATAGGGAGAGAGAGGAAAACCCACCTTCAAAAGGTG |
| 7991 | GGAGTTAATACGACTCACTATAGGGAGAGAGAGACCAAGCGGAATTACGTTGG |
