# Supplementary material for: Blocking tombusvirus replication through the antiviral functions of DDX17-like RH30 DEAD-box helicase
Source: PLoS Pathog. 2019 May 28;15(5):e1007771. doi: 10.1371/journal.ppat.1007771 (PMC6555533; doi:10.1371/journal.ppat.1007771)
Supplement: S1 Fig — (DOCX) [file ppat.1007771.s003.docx]

**S1 Figure. Comparison of the conserved F position in the helicase core domain in the yeast Ded1 and the Arabidopsis RH30 DEAD-box helicases.**

CLUSTAL O(1.2.4) multiple sequence alignment

RH30 MSSYDRRFADPNSYRQRSGAPVGSSQPMDPSAAPYNPRYTGGGGGYGPSPVMAGDNSGYN 60

DED1 MAELSEQVQN-------------------------LSINDNNENGYVPPHLRGKPRSARN 35

*:. ..:. : .. .** * : . .*. *

RH30 RYPSFQPPSGGFSVGRGGG-------RGGYG---QYGDRNGGGNWGGGGGRGGSSKRELD 110

DED1 NSSNYNNNNGGYNGGRGGGSFFSNNRRGGYGNGGFFGGNNGGSRS-----NGRSGGRWID 90

. .:: .**:. ***** ***** :*..***.. .* *. * :*

RH30 SV--SLPKQNFGNLVHFEKNFYVESPTVQAMTEQDVAMYRTERDISVEGRDVPKPMKMFQ 168

DED1 GKHVPAPRNEKAEIAIFGV---PEDPNFQSS-GINFDN-YDDIPVDASGKDVPEPITEFT 145

. *::: .::. * *.*..*: :. : :...*:***:*:. *

RH30 DANFPDNILEAIAKLGFTEPTPIQAQGWPMALKGRDLIGIAETGSGKTLAYLLPALVHVS 228

DED1 SPPLDGLLLENIKLARFTKPTPVQKYSVPIVANGRDLMACAQTGSGKTGGFLFPVLSESF 205

. : . :** * **:***:* . *:. :****:. *:****** .:*:*.* .

RH30 AQP----------RLGQDDGPIVLILAPTRELAVQIQEESRKFGLRSGVRSTCIYGGAPK 278

DED1 KTGPSPQPESQGSFYQRKAYPTAVIMAPTRELATQIFDEAKKFTYRSWVKACVVYGGSPI 265

:. * .:*:*******.** :*::** ** *:: :***:*

RH30 GPQIRDLRRGVEIVIATPGRLIDMLECQHTNLKRVTYLVLDEADRMLDMGFEPQIRKIVS 338

DED1 GNQLREIERGCDLLVATPGRLNDLLERGKISLANVKYLVLDEADRMLDMGFEPQIRHIVE 325

* *:*::.** ::::****** *:** : .* .*.********************:**.

RH30 QIR----PDRQTLLWSATWPREVETLARQFLRDPYKAIIGSTDLKANQSINQVIEIVPTP 394

DED1 DCDMTPVGERQTLMFSATFPADIQHLARDFLSDYIFLSVGRVGSTS-ENITQKVLYVENQ 384

: :****::***:* ::: ***:** * :* .. .: :.*.* : * .

RH30 EKYNRLLTLLKQLMDGSKILIFVETKRGCDQVTRQLRMDGWPALAIHGDKTQSERDRVLA 454

DED1 DKKSALLDLLSASTDG-LTLIFVETKRMADQLTDFLIMQNFRATAIHGDRTQSERERALA 443

:* . ** **. ** ******** .**:* * *:.: * *****:*****:*.**

RH30 EFKSGRSPIMTATDVAARGLDVKDIKCVVNYDFPNTLEDYIHRIGRTGRAGAKGMAFTFF 514

DED1 AFRSGAATLLVATAVAARGLDIPNVTHVINYDLPSDVDDYVHRIGRTGRAGNTGLATAFF 503

*:** : ::.** *******: ::. *:***:*. ::**:********** .*:* :**

RH30 THDNAKFARELVKILQEAGQVVPPTLSALVRSS-G------SGYGGSGGGRNFRPRGGGR 567

DED1 NSENSNIVKGLHEILTEANQEVPSFLKDAMMSAPGSRSNSRRGGFGRNNNRDYRKAGGAS 563

. :*:::.: * :** **.* ** *. : *: * * * ...*::* **.

RH30 GGGFGDKRSRSTSNFV-----PHGGKRTW------------ 591

DED1 AGGWGSSRSRDNSFRGGSGWGSDSKSSGWGNSGGSNNSSWW 604

.**:*..***..* .. . *
